# Stress resilience is an active and multifactorial process manifested by structural, functional, and molecular changes in synapses

**DOI:** 10.1016/j.ynstr.2024.100683

**Published:** 2024-10-22

**Authors:** E. Bączyńska, M. Zaręba-Kozioł, B. Ruszczycki, A. Krzystyniak, T. Wójtowicz, K. Bijata, B. Pochwat, M. Magnowska, M. Roszkowska, I. Figiel, J. Masternak, A. Pytyś, J. Dzwonek, R. Worch, K.H. Olszyński, A.D. Wardak, P. Szymczak, J. Labus, K. Radwańska, P. Jahołkowski, A. Hogendorf, E. Ponimaskin, R.K. Filipkowski, B. Szewczyk, M. Bijata, J. Włodarczyk

**Affiliations:** a Nencki Institute of Experimental Biology, Polish Academy of Sciences, Pasteur 3, Warsaw, 02-093, Poland; b Department of Regenerative Medicine, Maria Sklodowska-Curie National Research Institute of Oncology, Roentgena 5, Warsaw, 02-781, Poland; c AGH University of Krakow, Faculty of Physics and Applied Computer Science, Department of Medical Physics and Biophysics, al. A. Mickiewicza 30, 30-059, Krakow, Poland; d Maj Institute of Pharmacology, Polish Academy of Sciences, Smętna 12, 31-343, Cracow, Poland; e Mossakowski Medical Research Institute, Polish Academy of Sciences, Pawinskiego 5, 02-106, Warsaw, Poland; f Institute of Theoretical Physics, Faculty of Physics, University of Warsaw, Pasteura 5, 02-093, Warsaw, Poland; g Cellular Neurophysiology, Center of Physiology, Hannover Medical School, Carl-Neuberg-Str. 1, 30625, Hannover, Germany; h NORMENT Centre, Division of Mental Health and Addiction, Oslo University Hospital and Institute of Clinical Medicine, University of Oslo, Kirkeveien 166, 0424, Oslo, Norway

**Keywords:** *Stress*-*resilient animals*, *Dendritic spines*, *Palmitoylation*, *Chronic unpredictable stress*, *Behavioral stress response*

## Abstract

Stress resilience is the ability of neuronal networks to maintain their function despite the stress exposure. Using a mouse model we investigate stress resilience phenomenon. To assess the resilient and anhedonic behavioral phenotypes developed after the induction of chronic unpredictable stress, we quantitatively characterized the structural and functional plasticity of excitatory synapses in the hippocampus using a combination of proteomic, electrophysiological, and imaging methods. Our results indicate that stress resilience is an active and multifactorial process manifested by structural, functional, and molecular changes in synapses. We reveal that chronic stress influences palmitoylation of synaptic proteins, whose profiles differ between resilient and anhedonic animals. The changes in palmitoylation are predominantly related with the glutamate receptor signaling thus affects synaptic transmission and associated structures of dendritic spines. We show that stress resilience is associated with structural compensatory plasticity of the postsynaptic parts of synapses in CA1 subregion of the hippocampus.

## Teaser

**One Sentence Summary:** Compensatory remodeling of dendritic spines at the structural and molecular levels underlies stress resilience.

## 1 Introduction

The resilience phenomenon has been broadly investigated in physics (Suresh, 1998; Kakani, 2004; Hassler and Kohler, 2014), geoscience (Weichselgartner and Kelman, 2015), botany (Rankenberg et al., 2021), ecology (Pushpalal and Suzuki, 2020a), sociology (Adger, 2000; Bryan et al., 2019), economics (Plummer and Armitage, 2007), and neuroscience (Richter-Levin et al., 2021a; Stern et al., 2020). The definition of resilience is not universal and is often determined by researchers based on their own scientific interests (Richter-Levin et al., 2021a; Richardson, 2002; Den Hartigh and Hill, 2022; Fletcher and Sarkar, 2013; Southwick et al., 2014). The common feature of resilience manifests in its dynamic nature, i.e., the ability of a system (e.g., a material, a plant, or a brain) exposed to a harmful stimulus to absorb, accommodate or adapt to the effects of the stress in an efficient manner by adjusting its structure, organization, physiological mechanisms, metabolic regulation, mode of action, etc. The resilience phenomenon is also commonly observed in human neurological diseases, particularly in neurodegenerative and neuropsychiatric diseases (Stern et al., 2020; Arenaza-Urquijo and Vemuri, 2018; Hess et al., 2021; Davydov et al., 2010; Kane et al., 2019; Mizuno et al., 2016). Despite exposure to traumatic events or even to the presence of advanced pathophysiological changes, some people do not exhibit behavioral and psychological symptoms. The origin of such a diverse response to stressful conditions at the level of brain plasticity is an unexplored field of modern neuroscience. Many researchers point to the principal role of genetic predispositions in stress resilience, while others see its origin in protective factors, reward system, brain reserve or aberrant functions of key molecules (Wu et al., 2013; Peñ et al., 2019; Krishnan et al., 2007; Isingrini et al., 2016; Cathomas et al., 2019; Highland et al., 2019; Friedman et al., 2016; Stainton et al., 2019). Despite extensive research in this field, the impact of environmental factors on stress resilience remains largely unexplored, while chronic stress is widely assumed to be a key contributor in the pathogenesis of depressive phenotypes (Duman et al., 2016; Schmitt et al., 2014). The diverse distribution of glucocorticoid receptors within brain regions reveals the limbic regions (medial prefrontal cortex, hippocampus, amygdala) as the most affected structures by chronic stress in stress-related disorders (Duman et al., 2016; Schmitt et al., 2014). Several findings indicate that stress resilience can be pharmacologically enhanced upon treatment with glutamate receptor antagonists, e.g., ketamine (Highland et al., 2019; Krzystyniak et al., 2019; Brachman et al., 2016; Okine et al., 2020; Camargo et al., 2021). However, the changes in the molecular landscape underlying drug-induced stress resilience have not yet been identified. Of importance, in stress resilience research the depressive-like behavior defined by anhedonia underlies the behavioral reference for resilient phenotype results. Furthermore, the comprehensive, interdisciplinary characterization of the hippocampus has shown that the restoration of stress-related symptoms necessitates structural and functional excitatory plasticity in this brain region (Castré et al., 2021; MacQueen and Frodl, 2011; Holmes et al., 2019; Marsden, 2013).

The changes in structural connectivity are related to the processes occurring in the excitatory synapses (Sala and Segal, 2014a). Most of the excitatory synapses are located on dendritic spines, which are small, motile membrane protrusions of neurons (Tø et al., 2016). Dendritic spine remodeling involves alterations in spine morphology and/or density constituting a hallmark of physiological (learning and memory) and pathological (neurological and neuropsychiatric disorders) conditions (Penzes et al., 2011; Chidambaram et al., 2019; Bączyńska et al., 2021; Sala and Segal, 2014b). Studies of depressive-like behavior in animal models revealed abnormalities in the maintenance of the strength of synaptic connections that were manifested by a loss of spines and/or an increase in the proportion of immature, thin forms of spines. This finding suggests that the mechanism responsible for the transformation of dendritic spines might be a key factor underlying stress resilience (Bączyńska et al., 2021; Beyeler, 2019; Moda-Sava et al., 2019).

Recently, S-palmitoylation (S-PALM) has gained attention due to its role in stress-related neuropsychiatric diseases (Zaręba-Kozioł et al., 2018) and the remodeling of dendritic spines (Ji and Skup, 2021; Piguel et al., 2019; Fukata and Fukata, 2010). S-PALM is a fast-acting posttranslational lipid modification in which a palmitoyl group is attached to cysteine residues in peptides and proteins. S-PALM is one of the most unique posttranslational modifications because, unlike other lipid modifications, S-PALM is reversible (Mumby, 1997; Milligan et al., 1995) and may be regulated by the neuronal activity triggered by environmental factors (Fukata and Fukata, 2010) or pharmacological treatment (Piguel et al., 2019). S-PALM controls protein stability, receptor trafficking, and protein–protein interactions and thus contributes to synaptic plasticity, e.g., modulation of long-term potentiation (LTP) (Ji and Skup, 2021; Fukata and Fukata, 2010; Kang et al., 2008; Jeyifous et al., 2016). However, the role of S-PALM in stress resilience and the contribution of S-PALM to the dendritic spine remodeling is still an open subject and requires further study.

In the present study, we address the following question: is it possible to explain the potential differences in underlying synaptic plasticity between resilient and anhedonic animals in terms of the palmitoylation of synaptic proteins?

To answer the aforementioned question, we applied chronic unpredictable stress (CUS) leading to the development of resilient and anhedonic behavior. The evaluation of the functional, structural, and molecular readouts of excitatory synaptic plasticity in the hippocampus, in relation to animal behavior, was performed by a multidisciplinary and quantitative methodological approach that include mass spectrometry, electrophysiology, and fluorescent confocal imaging. The presented results indicate that stress resilience is a multifactorial phenomenon that actively develops during adulthood leading to aberrant synaptic plasticity. Moreover, we demonstrate that the developed stress-resilient state is associated with altered palmitoylation profile underlying structural, functional and molecular changes in excitatory synapses in the hippocampus.

## 2 Results

### 2.1 Chronic stress leads to the development of stress resilience and anhedonia in adult mice

**Fig. 1:**
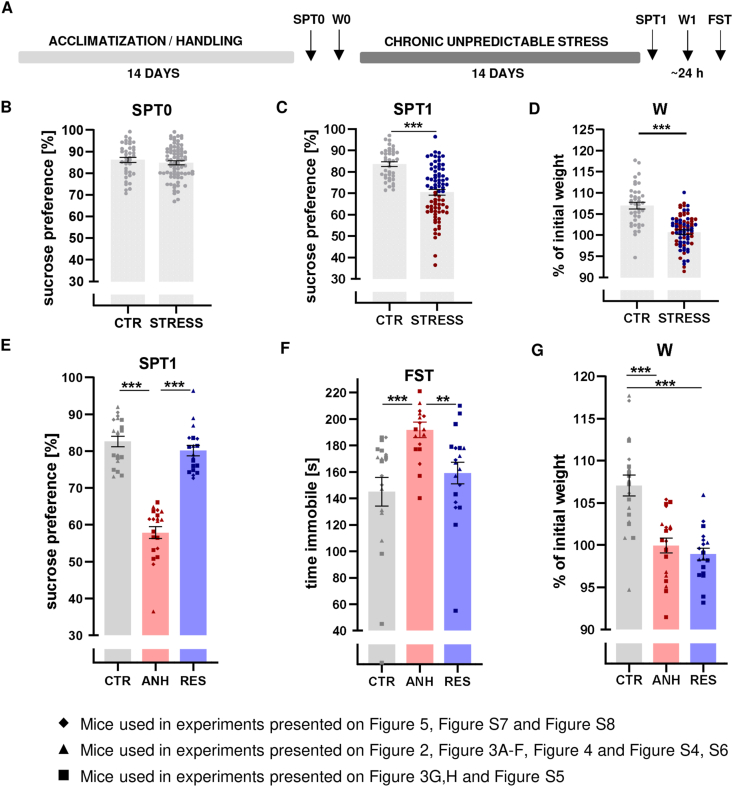
**Behavioral evaluation of depressive-like behavior of anhedonic (ANH) and resilient (RES) animals following chronic unpredictable stress (CUS) as well as control animals (CTR). (A)** Schematic view of the experimental design of the CUS model. **(B)** Baseline sucrose preference test (SPT0). **(C)** Sucrose preference test after CUS (SPT1), anhedonic animals are marked in red and resilient animals in blue **(D)** Body weight gain after CUS (W) n_CTR_ = 39, n_STRESS_ = 78 **(E**–**G)** Behavioral parameters of animals used in experiments described in the next figures n = 18–20. **(E)** Sucrose Preference Test after CUS (SPT1). **(F)** Forced swim test after CUS (FST). **(G)** Body weight gain after CUS (W). The data are presented as the mean ± SEM. ∗p < 0.05; ∗∗∗p < 0.001. (B-C – two tailed *t*-test, D - Mann Whitney test E-F-Dunn's multiple comparisons test, G – Brown-Forsythe ANOVA test F (2.000, 45.96) = 20.93 followed by Tamhane's T2 multiple comparisons test. (For interpretation of the references to color in this figure legend, the reader is referred to the Web version of this article.)

To investigate whether stress resilience might develop during adulthood, we employed CUS to induce a behavioral stress response in adult animals. We demonstrated that the implementation CUS procedure leads to the development of anhedonic and resilient behaviors, as determined by the sucrose preference test (SPT, Fig. 1A–C). After CUS, approximately 50% of mice developed anhedonic behavior, and the rest were classified as stress-resilient (Fig. 1C) (Bijata et al., 2022a, 2022b). Moreover, mice subjected to CUS (both anhedonic and resilient) exhibited less robust body weight gain than that of the non-stressed control animals. Thus, body weight is a physiological indicator of exposure to chronic stress (Fig. 1D) (Retana-Má et al., 2003; Tamashiro et al., 2011). Taken together, our data indicate that implementation of the designed CUS procedure leads to the synchronic development of anhedonic and resilient behavior in genetically homogenous adult mice, constituting a promising animal model of stress resilience. Fig. 1E–G and S1A-B shows the behavioral parameters (SPT, FST) and body weight gain of the mice that were used in experiments described in the next parts of manuscript. We observed a significant negative correlation between the SPT and FST results, although the Pearson correlation coefficient does not perfectly fit every data point (Fig. S1C, p = 0.02). Interestingly, the results obtained at 10 and 18 days after chronic stress indicate that anhedonic animals recover after cessation of stress, while the resilient phenotype stabilizes. At 25 days after chronic stress, resilient animals showed an increase in sucrose preference (Fig. S2).

### 2.2 Chronic stress affects the expression of synaptic proteins in the hippocampus

**Fig. 2:**
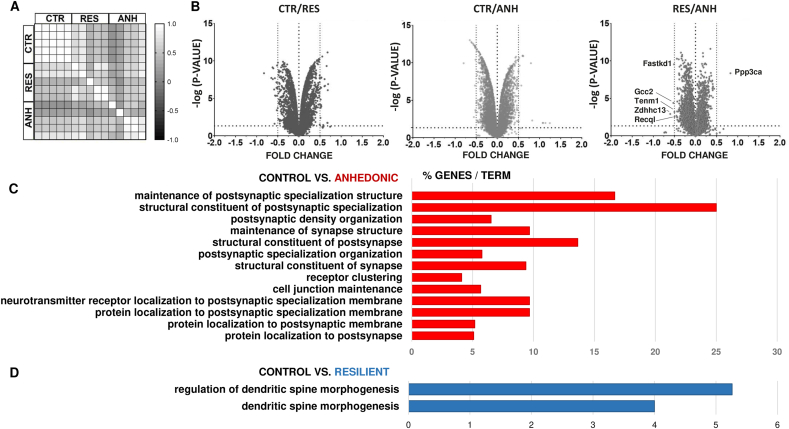
**Analysis of differentially expressed synaptic proteins from proteomic profiling. (A)** Matrix representation of Pearson correlation coefficients of protein abundances in 5 biological replicates. **(B)** Volcano plots representing changes in protein expression in CTR, ANH, and RES animals. The fold change log (base 2) is on the x-axis, and the negative false log discovery rate (p value, base 10) is on the y-axis. Negative fold change indicates increased expression while positive fold change decreased expression. (Two-stage linear step-up procedure of Benjamini, Krieger and Yekutieli). **(C**–**D)** Kyoto Encyclopedia of Genes and Genomes (KEGG) bioinformatics analysis of the significantly altered signaling pathways of the ANH and RES groups. The p value negative log (base 10) is on the x-axis.

As a first step to determine the molecular fingerprint of stress resilience, we employed a high-throughput proteomic approach using mass spectrometry to generate a comprehensive view of the *in vivo* proteins level in the hippocampal synaptoneurosomes of control, resilient and anhedonic animals. To isolate synaptoneurosomes we used a method based on ultracentrifugation and a density gradient (Zareba-Koziol et al., 2019; Zaręba-Kozioł et al., 2014). We evaluated the quality of the synaptoneurosome preparation using electron microscopy (Fig. S3). To identify differentially expressed proteins across behavioral groups, their protein levels were determined and comparatively analyzed in five biological replicates per group. Additionally, the clustering heatmaps of Pearson correlation coefficients (PCCs) of peptide signal intensity were determined using logarithm 2 transformed protein abundance data (Fig. 2A) to evaluate the variability in the proteomic analysis within biological replicates and behavioral groups. The correlation matrix shows the PCC values for all experimental groups. The matrix values for each stressed group differed from the matrix values for the control group. Moreover, the variation in the matrix values for the control group was small, in contrast to the variations for the other two groups (Fig. 2A). Volcano plots depict the changes in protein expression among behavioral groups (Fig. 2B). The fold change logarithm (base 2) is on the x-axis, and the negative logarithm of the false discovery rate (p value) (base 10) is on the y-axis. Protein levels with p value < 0.05 and fold changes < −0.5 and >0.5 were considered significantly different. We identified 6224 proteins with less than 1% FDR (false discovery rate). Our results revealed 44 differential synaptic proteins in resilient animals (24 upregulated and 20 downregulated) and 39 in the anhedonic group (20 upregulated and 19 downregulated) in comparison to non-stressed animals. The list of the proteins within the aforementioned groups is presented in Supplemental Tables 1A–B. Surprisingly, the levels of only 6 synaptic proteins significantly differed between the resilient and anhedonic groups: Ppp3ca, Recql, Tenm1, Fastkd1, Gcc2 and Zdhhc13 (one upregulated and five downregulated; for more details, see Supplemental Table 1C, Fig. 2B). Altogether, chronic stress affected less than 1% of the identified synaptic proteins in the hippocampus. In KEGG pathway bioinformatics analysis we found that the most significantly enriched pathways in anhedonic mice were related to maintenance of postsynaptic specialization, postsynaptic density organization, structural constituent of postsynapse, receptor clustering, neurotransmitter receptor localization to postsynaptic specialization membrane and protein localization to postsynapse (Fig. 2C). In resilient mice the most affected pathways were related to regulation of dendritic spines morphogenesis (Fig. 2D).

### 2.3 Distinctive palmitoylation of synaptic proteins in stress resilience

**Fig. 3:**
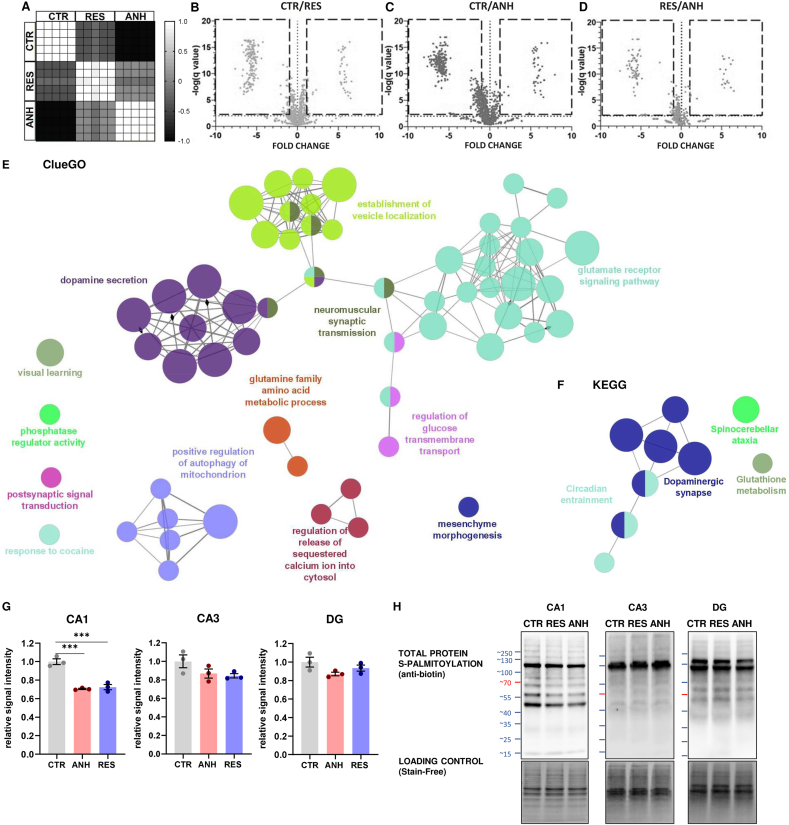
**Analysis of differentially S-palmitoylated synaptic proteins from proteomic profiling. (A)** Matrix representation of Pearson correlation coefficients of protein abundances in 5 biological replicates. **(B**–**D)** Volcano plots represent changes in protein S-palmitoylation in hippocampal synaptoneurosomes of CTR, ANH, and RES animals. The fold change log (base 2) is on the x-axis, and the negative false log discovery rate (p value) (base 10) is on the y-axis (Two-stage linear step-up procedure of Benjamini, Krieger and Yekutieli). **(E)** ClueGO, and **(F)** KEGG bioinformatics analysis of S-PALM proteins characteristic of stress resilience. Functionally grouped networks are linked to their GO biological processes and KEGG pathways. Each circle (node) represents a biological term consisting of various related proteins/genes. The node size represents the enrichment significance. Terms that belong to the same pathway are marked with the same color, and terms associated with two different pathways are marked with two colors. The connectivity (edges) between the terms in the network is derived from the kappa score (indicates the similarity of associated genes shared by different terms). Thicker edges indicate stronger similarity. Diamonds represent directed edges that link parent terms to child terms. Only the name of the most significant term in each group is shown to reduce the overlay. **(G)** Total palmitoylation of proteins in different subregions of the hippocampus (CA1, CA3, DG) of CTR, ANH, and RES animals. The experiment was performed in triplicate, using pooled samples from three independent animals per each condition (n = 9) The data are presented as the mean ± SEM; ∗∗p < 0.01; ∗∗∗p < 0.001 (one-way ANOVA followed by Sidak's multiple comparisons test, CA1 F (2, 6) = 46.65; CA3 F (2, 6) = 2.674; DG F (2, 6) = 2.857). (For interpretation of the references to color in this figure legend, the reader is referred to the Web version of this article.)

Due to the potential role of palmitoylation in chronic stress and neuropsychiatric diseases, our special attention was drawn to the palmitoyltransferase Zdhhc13 (DHHC22) (Zareba-Koziol et al., 2019; Gorinski et al., 2019). We hypothesized that the altered level of palmitoyltransferases expression may alter the S-palmitoylation (S-PALM) profile of synaptic proteins differentially in stress resilient and anhedonic animals. We therefore took an unbiased proteomic approach based on the mass spectrometry PANIMoni method (biotin labeling of S-palmitoylated proteins) (Zareba-Koziol et al., 2019) to identify proteins palmitoylated in response to the CUS procedure. We examined the level of similarity of protein content within the groups using principal component analysis (PCA) and clustering heatmaps of PCCs. We confirmed the reproducibility of the proteome preparation, enabling us to observe a global distinction between the control and stressed mice in the S-PALM profile of synaptic proteins. The results of the relative quantification experiment are summarized in Fig. 3A–D and Supplemental Table 2A. We identified S-PALM of 1199 synaptic proteins upon the CUS procedure in which 113 proteins were commonly changed in the same direction with a fold change < −1 and >1 and p < 0.01 for all stressed mice, while 188 of synaptic protein differentiated the resilient phenotype from those of the anhedonic and control animals, constituting the S-PALM fingerprint of stress resilience (Supplemental Table 2B). To determine which cellular and synaptic processes were involved in stress resilience-induced turnover of palmitoylation, we analyzed the datasets of differentially palmitoylated proteins using the synaptic protein database ClueGO. In the ClueGO analysis, we identified functionally grouped networks linked to their GO biological processes and KEGG pathways, indicating palmitoylation-dependent biological processes (Fig. 3E–F) that were associated mostly with the glutamate receptor signaling pathway (33.33% of terms, e.g., Daglb, Frrs1, Grin2b, Plcb1, Ptk2b, Shank3, Crtc1, Epha4, Rab3a,Tbc1d24), among them processes such as dendritic spine morphogenesis (e.g., Epha4, Kif1a, Shank3, Tanc2, Ptk2b, Tbc1d24, Erc1, Rab3a), receptor clustering (e.g., Grin2b, Lrp4, Shank3), synaptic vesicle turnover and localization (e.g., Ap3d1, Ap3m2, Htt, Kif1a, Rab3a, Shroom2, Tanc2, Ykt6, Tbc1d24, Ppp3cb, Fig. S4) and behavioral fear response (e.g., Grin2b, Shank3, Vdac1, Fig. S4). The other abundant functional cluster was found to be dopamine secretion (15.94 % of associated terms, e.g., Abat, Kcna2, Prkcb, Rab3a, Syt3). In addition, to address whether S-PALM is differentially manifested in different subregions of the hippocampus, the total S-PALM levels of all proteins were determined using acyl-biotin exchange method (Fig. 3G–S5). Obtained results indicate that chronic stress leads to decrease in S-PALM level mainly in the CA1 subregion of the hippocampus in comparison to the CA3 and dentate gyrus (DG) subregions.

### 2.4 Chronic stress impairs postsynaptic glutamatergic neurotransmission in the hippocampus

Since we have observed changes in the palmitoylation profile of proteins related with the glutamate receptor signaling pathway in the hippocampus after chronic stress in resilient animals, we studied synaptic transmission by recording field excitatory postsynaptic potentials (fEPSPs) in acute hippocampal brain slices. To determine the α-amino-3-hydroxy-5-methyl-4-isoxazolepropionic acid receptors (AMPARs) and *N*-methyl-D-aspartate receptors (NMDARs) that contributed to synaptic transmission, we analyzed fEPSPs evoked in response to monotonically increased stimuli in the CA3-CA1 hippocampal projections in magnesium-free artificial cerebrospinal fluid (aCSF, Figs. S6A–B) (Brzdą et al., 2017). Experimental data were fitted with mathematical functions and compared for statistically significant differences by means of Monte Carlo randomization (see Materials and Methods, for details). Input–output curves in CA1 region showed that the amplitudes of the AMPAR-mediated fEPSPs as wells as the area of the NMDAR-mediated component of fEPSPs were not significantly different between groups (Figs. S6C–D). We next studied the CA3 region and recorded responses of associational-commisural synapses in response to retrograde stimulation of the CA3 subregion (Wójtowicz et al., 2016). We found that similar to CA1, CUS in the CA3 region resulted in no significant alteration of fEPSP amplitudes (Fig. S6A). In contrast, the area of the NMDAR-mediated component of fEPSPs in CA3 region was significantly upregulated in ANH compared to RES group (p = 0.031) while other comparison did not reveal significant differences (Monte-Carlo simulations, Fig. S6). Thus, CUS resulted in differential modulation of glutamatergic AMPAR- and NMDAR-mediated synaptic responses in hippocampal subregions.

**Fig. 4:**
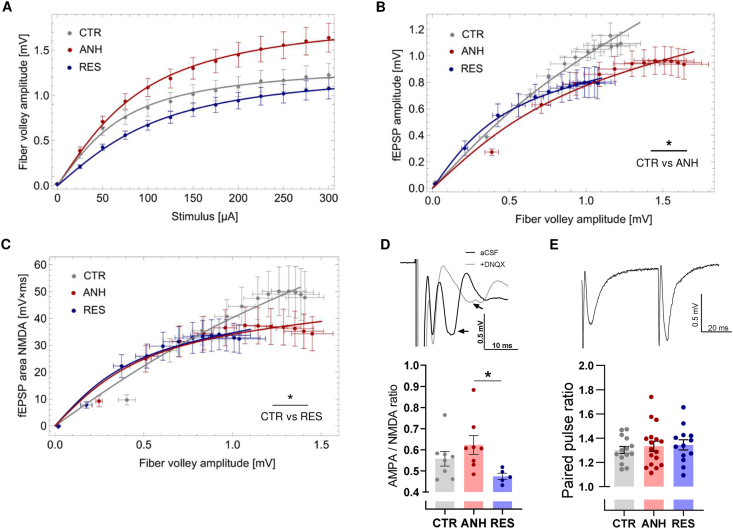
**Chronic stress promotes differential changes in glutamatergic neurotransmission in ANH and RES hippocampi. (A**–**C)** Average input–output relationships for fEPSPs recorded in the CA1 subregion of acute hippocampal slices and evoked in response to monotonically increased current stimuli applied to Shaffer collaterals. **(A)** fiber volley amplitudes were not significantly different following CUS but tended to vary between animal groups(p > 0.05, n_slices_ = 15–20, N_animals_ = 5–6 per group, experimental data were fitted with a mathematical function and compared for statistically significant differences by means of three-dimensional Monte Carlo simulations (see Materials and Methods, for details). Experimental data were fitted with the function y(x) = a arctgx/b; see Materials and Methods for details). **(B**–**C)** fEPSP amplitudes and fEPSP area values normalized to fiber volley amplitudes in the same recordings (n_slices_ = 12–20, N_animals_ = 5–6 per group). **(B)** The magnitude of AMPAR-mediated fEPSPs recorded for the same fiber volley amplitude was significantly lower in the ANH but not the RES compared to the CTR group (p = 0.03 and p = 0.25 respectively). There was no difference between ANH and RES groups (p = 0.54). **(C)** the NMDAR-mediated fEPSPs were significantly lower in RES group compared to CTR (p = 0.01) while ANH vs RES and ANH vs CTR did no differ (p = 0.055 and p = 0.61, respectively). **(D**) Top panel shows exemplary traces of compound AMPAR/NMDAR-mediated fEPSPs in Mg^2+^-free aCSF before (black) and after application of AMPAR antagonist DNQX (gray). recorded in response to paired-pulse stimulation. Arrows indicate a change in the peak fEPSP amplitude. Lower panel shows statistics of these recordings. The ANH group exhibited a higher AMPAR/NMDAR ratio estimated after DNQX application compared to that of the RES group (n_slices_ = 5–8, N_animals_ = 4–6 per group, F(2,18) = 3.664, p = 0.04, one-way ANOVA, see Materials and Methods for details). **(E)** Top panel shows exemplary traces of fEPSPs recorded in response to paired-pulse stimulation. Lower panel shows statistics of these recordings. CUS did not affect the paired-pulse facilitation ratio (n_slices_ = 13–17, N_animals_ = 5–6 per group, F(2,39) = 1.514, p = 0.23, One-way ANOVA)). Data are presented as the mean ± SEM. ∗p < 0.05.

To have further insight into this phenomenon, we have studied more thoroughly CA1 excitatory synapses. Surprisingly, we observed the largest apparent difference in the amplitudes of the fiber volley, although these differences were not significantly different (p > 0.05, Monte Carlo method, Fig. 4A). The large variance in average fiber volley amplitudes could reflect changes in presynaptic axon function and/or the number of available afferent fibers following chronic stress (Kim et al., 2012). Therefore, we visualized the fEPSP amplitude and the area vs. respective fiber volley amplitudes preceding these synaptic responses (Fig. 4B–C). We found that the magnitude of AMPAR-mediated fEPSPs recorded for the same fiber volley amplitude was significantly lower in the ANH but not the RES compared to the CTR group (Fig. 4B). In addition, the NMDAR-mediated fEPSPs were significantly lower in RES group compared to CTR (Fig. 4C). Therefore, stimulation of the same number of presynaptic afferents yielded less efficient excitatory AMPAR-mediated synaptic drive in the anhedonic group and NMDAR-mediated synaptic transmission in resilient group.

Consequently, we investigated whether chronic stress is associated with an altered synaptic AMPAR/NMDAR ratio. To this end, we compared the sensitivity of compound fEPSPs to an AMPAR and kainate receptor antagonist (DNQX), as described previously (Brzdak et al., 2019). We found a larger sensitivity to DNQX, and thus lower AMPAR/NMDAR ratio in the resilient group than in the anhedonic group (Fig. 4D). The potentiation of synaptic release in response to paired-pulse stimulation was similar among the investigated groups (Fig. 4E). Therefore, an altered presynaptic release was not attributed to any behavioral phenotype. Taken together, chronic stress affects glutamatergic neurotransmission in the hippocampus differently in the anhedonic group (less efficient excitatory drive and an increased AMPAR/NMDAR ratio) than in the resilient group. Since the observed functional alterations in the CA1 subregion of the hippocampus were limited to the postsynaptic part of the excitatory synapses, we subsequently analyzed the structural features of dendritic spines.

### 2.5 Stress resilience is associated with structural compensation in the hippocampus

**Fig. 5:**
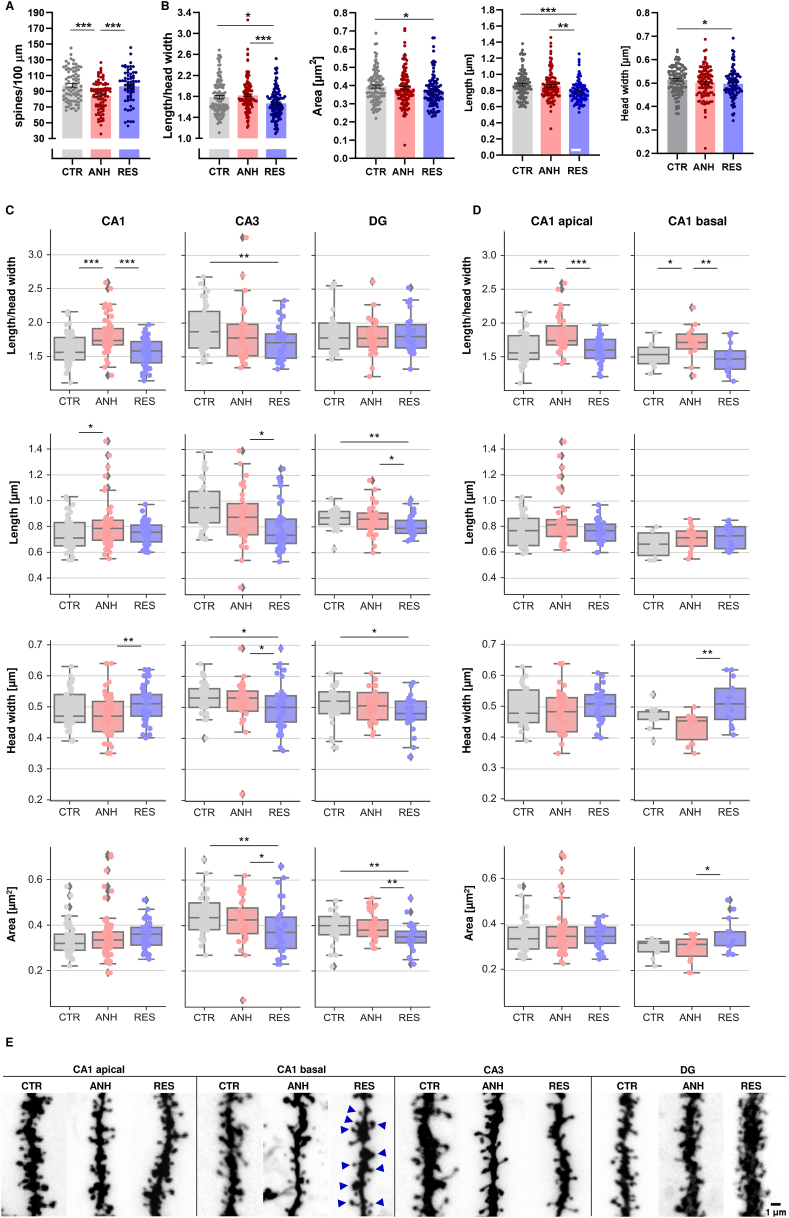
**Chronic stress differentially affects the density and morphology of dendritic spines in the hippocampus of CTR, ANH, RES animals,** including **(A)** spine density, **(B)** morphology in scale-free parameter (length/head width ratio) of relative morphometric changes, dendritic spine projected area, projected length, head widthpooled data from the entire hippocampus including data from CA1 (basal and apical dendrites), CA3, and DG. CTR N_spines_ = 7735, N_dendrites_ = 116, N_animals_ = 5; ANH N_spines_ = 7918, N_dendrites_ = 120 N_animals_ = 5; RES N_spines_ = 7026 N_dendrites_ = 107, N_animals_ = 5, **(C)** Morphometric analysis of dendritic spines in the CA1 (including apical and basal dendrites), CA3, and DG subregions of the hippocampi of CTR, ANH, RES animals **(D)** Morphometric analysis of dendritic spines localized in the apical and basal dendrites in the CA1 subregion of the hippocampi of CTR, ANH, RES animals **(E)** representative confocal images of CTR, ANH, RES hippocampal dendritic segments. The arrows indicate the mushroom-shaped dendritic spines in the RES group. Scale bar = 1 μm. Data are presented as the mean ± SEM. The points on the graphs represent the dendrite fragments in 1 field of view; ∗p < 0.05; ∗∗p < 0.01, ∗∗∗p < 0.001 (nested ANOVA).

To determine how chronic stress affects dendritic spine structure, we performed DiI staining on hippocampal slices obtained from the second hemisphere and visualized them by fluorescence confocal microscopy. In the analysis of the hippocampus, we observed that the resilient animals differed from the control animals with respect to dendritic spine morphology in glutamatergic neurons. The spine density of the resilient animals was unchanged, while in the anhedonic group, decreased spine density was observed (p < 0.0001, Fig. 5A–E, Fig. S9). The quantitative analysis of spine morphometry in the entire hippocampus showed a decreased *length-to-head-width* ratio and spine projected *area* in the resilient group than in the controls (p < 0.05, Fig. 5B–E, Fig. S9). Upon analysis of the individual subregions (i.e., pyramidal neurons localized CA1, CA3 and granule neurons in DG), we identified morphological changes unique to a given subregion. In the CA3 subregion, the spines of RES animals showed a lower length-to-head-width ratio compared to that of CTR animals. Such changes usually indicate the maturation of the dendritic spines, however a more detailed morphometric analysis revealed that the changes in the length-to-head-width ratio were mainly due to a decrease in surface area and head width which may indicate pathological changes resulting from chronic stress (Fig. 5C–E, Figs. S8 and S9). However, the CA1 subregion caught our special attention. In this subregion, the spines of ANH animals showed a higher *length-to-head-width* ratio compared to CTR animals and RES animals. In parallel, the spines of RES animals had a larger head width than that of anhedonic animals, and there was a clear upward trend compared to CTR animals (p = 0.10) (Fig. 5C–E, Figs. S8 and S9). In the CA1 subregion pyramidal neurons develop two dendritic trees covered with dendritic spines - basal dendritic tree located in *Stratum Oriens* and apical dendritic tree in *Stratum Radiatum*. In addition to the different neuroanatomical distribution, these trees differ in their molecular composition, e.g., the protein palmitoylation profile (Brzdak et al., 2019), therefore we decided to perform a separate morphometric analysis of dendritic spines located on the basal dendritic tree and apical dendritic tree in the CA1 subregion of the hippocampus. Morphometric analysis revealed that in ANH animals in both basal and apical dendritic trees there was an increase in *length-to-head-width* ratio in comparison to control animals and RES animals (Fig. 5D–E, Fig. S8, SS9). On the other hand, only in the basal dendritic tree in RES animals we observed a trend in the decrease in *length-to-head-width* ratio in comparison to control animals (p = 0.20) (Fig. 5D–E, Figs. S8 and S9). Moreover, a detailed analysis showed that changes in *length-to-head-width* ratio of RES animals in the basal dendritic tree are associated with a significant increase in the *head width* and the projected *area* of the dendritic spine in comparison to control animals and ANH animals (Fig. 5D–E, Figs. S8 and S9). This result indicates maturation of dendritic spines in this subregion in RES animals, which may be the basis of the mechanism of structural compensation in RES animals. In summary, we conclude that resilient behavior is accompanied by structural compensation in the hippocampus, which is shown in the representative confocal images of dendritic spines in Fig. 5E.

## 3 Discussion

In the present study, we aimed to investigate whether stress resilience is an actively-developed process in adult mice. Therefore, we determined the molecular fingerprint of stress-resilient behavior involved in hippocampal neuronal circuits upon chronic stress. We demonstrated that stress resilience is associated with alterations in glutamate receptor signaling pathways, which are exclusively limited to the postsynaptic parts of synapses. We showed that stress resilience manifests itself by compensatory remodeling of dendritic spines, combined with concomitant changes in the S-PALM of synaptic proteins involved in spine morphogenesis, receptor trafficking and glutamatergic neurotransmission.

The complex characterization of synaptic plasticity in resilient animals is an appealing issue. Most of the research on stress resilience is related to a behavioral evaluation in early-life maternal separation models or to the genetic knockouts of targeted proteins that promote stress-resilient behavior in adulthood (Rankenberg et al., 2021; Richter-Levin et al., 2021b; Pushpalal and Suzuki, 2020b). Therefore, we discuss our results with respect to animal models that share similar components of stress procedures to those based on the chronic unpredictable stress and social defeat paradigms. As a result of the multifaceted and comprehensive analysis of the characteristics of the resilient and anhedonic phenotypes, which differ from that of the non-stressed mice, we observed specific changes in synaptic plasticity within behavioral groups. Similar to reports in the literature (Tang et al., 2019; D et al., 2017; Han et al., 2015; McEwen et al., 2015), we observed significant differences in the expression levels of synaptic proteins following chronic stress, but those changes did not differentiate the resilient and anhedonic phenotypes. Interestingly, our analysis revealed that there are also no significant differences in total S-PALM between the ANH and RES groups. However, when examining the S-PALM of individual synaptic proteins, we observed significant differences between these two groups. These findings suggest that it is not global changes in palmitoylation, but rather specific modifications of palmitoylation of individual proteins or even specific cysteines that may play a significant role in differentiating resilience and anhedonia behaviors. This result is consistent with what we previously obtained when examining the palmitoylation of all proteins from PFC- while total S-PALM remained unchanged, we observed specific changes in the S-PALM of the 5-HT1A receptor in mice and rats exhibiting depressive-like behaviors as well as in individuals with MDD that died by suicide (Gorinski et al., 2019). Importantly, the ClueGO analysis revealed that significantly altered expression levels of the synaptic proteins associated with chronic stress were particularly associated with morphogenesis of dendritic spines and processes related to protein localization to the postsynaptic membrane, which is in agreement with the results of the aforementioned studies. Several authors noticed robust proteomic alterations in the hippocampus following chronic stress (Tang et al., 2019; Peri et al., 2018; Gottschalk et al., 2018). However, our results revealed more subtle changes than those reported earlier. This difference may be because we used the synaptoneurosomal fractions and not brain homogenates from the entire hippocampus.

Chronic stress has been shown to affect hippocampal glutamatergic neurotransmission as well as learning and memory (McEwen et al., 2016; Popoli et al., 2012; Yang et al., 2018a). In our study, we focused on the general characteristics of the AMPAR- and NMDAR-mediated fEPSPs in each behavioral group. We did not aim to assess how the chronic stress affects LTP and learning and memory in mice. Nevertheless, our CUS protocol was based on that in a study by Strekalova et al. (Strekalova et al., 2004; Strekalova and Steinbusch, 2010) in which behavioral evaluation for learning and memory was performed, showing cognitive impairments in anhedonic animals. This experimental direction is particularly worth investigating in the future, as studies in humans have shown that resilient individuals exhibit cognitive enhancement (Parsons et al., 2016; Miyagi et al., 2020). The potential cognitive enhancement in the resilient animals may arise from the structural compensation of dendritic spines in the hippocampus (manifested by the higher spine density and levels of the observed spine maturation of already existing spines than those in the anhedonic group). The importance of appropriate restoration of the density and morphology of dendritic spines is emphasized in strategies of MDD treatment (Penzes et al., 2011; Bączyńska et al., 2021; Moda-Sava et al., 2019; Pfarr et al., 2021). In particular, the results obtained in preclinical studies concerning the activity of fast and long-acting antidepressants, such as glutamate-based antidepressants, e.g., ketamine, or serotonin-based compounds, such as psilocybin, have shown that sustained antidepressant-like effects could causally depend on changes in the density and/or morphology of dendritic spines (Li et al., 2010; Ng et al., 2018; Shao et al., 2021). In general, not every type of stress protocol affects the structure of dendritic spines similarly. In fact, only severe and prolonged exposure leads to pathological remodeling of spines (density and/or morphology), which is assumed to be a structural correlate of depressive symptoms in rodents (Kim et al., 2015). In the present study, we have shown that anhedonic animals exhibited a decrease in spine density with no morphometric changes within the whole hippocampus. However, as in the previous study, we have demonstrated that anhedonic behavior is associated with spine elongation exclusively in the CA1 subregion of the hippocampus and that this subregion of the hippocampus is involved in the development of anhedonic behavior (Bijata et al., 2022a). The results obtained in the current study suggest that the CA1 subregion of the hippocampus is also affected by structural and molecular changes during resiliency. Going even deeper, our results suggest that chronic stress differentially affects the structural plasticity of the proximal (basal dendrites localized in *Stratum Oriens*) and distal (apical dendrites localized in *Stratum Radiatum*) areas of the hippocampal CA1 subregion. Interestingly, the proximal parts of the dendrites have been shown to have greater regenerative capacity compared to the distal dendrites (Goldwater et al., 2009; Radley et al., 2005), which is consistent with the observed different structural effects in the basal and apical dendritic trees of pyramidal cells after exposure to CUS. In addition, it is known that, apart from different neuroanatomical distribution, these trees differ in molecular and functional terms (Brzdak et al., 2019). In line with the results of other studies (Yang et al., 2018b; Suo et al., 2013; Xu et al., 2018), we did not observe differences in the expression levels of synaptic proteins belonging to the mTORC1 complex following chronic stress. Nevertheless, the reduced level of Rictor protein (mTORC2 binding partner) in the resilient group gained our attention due to mTORC2 insensitivity to the rapamycin (Sarbassov et al., 2004). The role of mTORC2 in the hippocampal synaptic plasticity has been widely reported (McCabe et al., 2020; Angliker et al., 2013; Zhu et al., 2018). However, how its complex may be engaged in the behavioral stress response and how it is palmitoylated have not yet been studied. Notwithstanding, we also observed differences in the S-PALM (Cys-162) of tetratricopeptide repeat ankyrin repeat and coiled-coil containing 2 (TANC2) between the resilient and anhedonic animals and the controls. Deletion of TANC2 in the hippocampus hyperactivates mTORC1/mTORC2-dependent signaling pathways, leading to cognitive impairment and hyperactivity in mice (Kim et al., 2021). Moreover, TANC2 directly interacts with postsynaptic density protein 95 (PSD-95) and constitutes an endogenous inhibitor of mTORC1/mTORC2 complexes (Kim et al., 2021). Moreover, it has been shown that ketamine activates the mTORC1 complex by suppressing the interaction of TANC1/2 with mTOR interaction but does not affect the interaction of TANC1/2 with PSD-95^95^. Despite the still unknown physiological role of S-PALM of TANC2 (Cys-162), its role in the biological basis of stress resilience should be considered as a potential molecular target in future studies.

In addition to the involvement of the mTOR complex in the regulation of dendritic spine structure, as well as glutamatergic neurotransmission, GSK-3-beta kinase also plays a decisive role in the development of depressive-like behavior, and the structural remodeling of dendritic spines (Li and Jope, 2010; Kondratiuk et al., 2017; Duda et al., 2020). In particular, our results have revealed the possible role of GSK-3-beta kinase in the genesis of resilient behavior. We observed a decreased level of beta-catenin (responsible for cell survival in a Wnt-dependent manner), suggesting the impact of GSK-3-beta/APC complex activity in the resilient group. The distinct S-PALM of two cysteines (Cys-912 and Cys-2664) in adenomatous polyposis coli (APC) protein was also observed in resilient and anhedonic mice. Due to the formation of a complex of APC and GSK-3-beta kinase, leading to the degradation of beta-catenin in the proteasome (Beurel et al., 2015), the S-PALM of APC might be involved in the regulation of beta-catenin levels. Moreover, we observed a decrease in the level of the aforementioned Rictor protein in the resilient group, which was negatively correlated with the functioning of GSK-3-beta kinase (Koo et al., 2015). Thus, we can speculate that enhanced S-PALM of APC can negatively regulate the formation of the APC/GSK-3-beta complex, decreasing the degradation of proteins, such as beta-catenin or Rictor, in the anhedonic phenotype. However interesting, these outcomes should be interpreted carefully. First, the level of beta-catenin is not regulated only by its degradation in the proteasome; therefore, the correlation between the expression of beta-catenin and the activation of the GSK-3-beta/APC complex could be misleading (Beurel et al., 2015). Concurrently, GSK-3-beta kinase activity is much more complicated than its simple involvement in the complex with APC (Beurel et al., 2015). In particular, the interplay between GSK-3-beta kinase and mTOR complexes, such as TSC-1/TSC-2, is not dependent on the GSK3-beta/APC complex but on the dephosphorylation of GSK-3-beta (Ser-9) (Beurel et al., 2015; Evangelisti et al., 2020). Therefore, a proper understanding the role of GSK-3-beta kinase in resilient and anhedonic mice requires more profound studies with complex profiling of the phosphorylation of synaptic proteins. Despite the unclear role of GSK-3-beta kinase in the decreased level of beta-catenin in the resilient phenotype, the role of this protein in the development of stress resilience is still interesting. Vidal et al. showed that inhibition of beta-catenin in GLAST-expressing cells lead to the development of depressive-like behavior while its stabilization led to a resilient state in a model of chronic exposure to corticosterone (Vidal et al., 2019). Therefore, the decreased level of beta-catenin in the hippocampus of resilient animals should be considered in the context of other signaling pathways because, as we showed, different cellular events underlie stress resilience and depressive-like behavior. The NMDAR and AMPA receptor subunits revealed alterations in S-PALM following chronic stress. In particular, we observed increased levels of the palmitoylation of the GluA1 subunit of AMPA receptors at two cysteines (Cys-601 and Cys844). As previously reported, palmitoylation of these cysteine residues increases the anchoring of the receptor GluA1 subunit to the Golgi apparatus and inhibits the interaction between the subunit and the synaptic 41N protein, affecting glutamatergic transmission (Hayashi et al., 2005; Lin et al., 2009). Increased palmitoylation of the Cys-202 residue of the GDP dissociation inhibitor-1 (GDI-1) exclusively in the anhedonic group may also indirectly regulate the turnover of AMPA receptors anchored to dendritic spines and may explain the observed decrease in spine density. It was shown previously (Wei et al., 2010; Hausser and Schlett, 2019) that the interactions of GDI-1 with the Rab family proteins are responsible for maintaining equilibrium between exocytosis and endocytosis through LTP. Unfortunately, the physiological role of Cys-202 palmitoylation of GDI-1 remains unknown. We also observed an increased level of palmitoylation of two cysteines (Cys-954 and Cys-1173) in the cytoplasmic domain of the GluN2B subunit in the anhedonic group. However, the physiological role of these GluN2B modifications has also not been described. Nevertheless, the differences in the functional readout of AMPA and NMDA receptors upon chronic stress could also be explained by the fact that palmitoylation is not the only posttranslational modification that occurs at the synapse. Several serine residues in the GluA1-A4 subunits of the AMPA receptor, as well as the GluN2B subunit, undergo phosphorylation, affecting receptor trafficking, conductance, and the frequency of the channel opening (Purkey et al., 2020; Lee et al., 2010; Lu et al., 2015). Therefore, the interplay between the expression, palmitoylation, phosphorylation, and ubiquitination of synaptic proteins produces a functional effect.

The mechanism underlying region-specific differences in the function of excitatory synaptic transmission in CA1 and CA3 regions in anhedonic and resilient animals remain elusive (Fig. 4, 6 and 7). At least in CA1 region, most likely the postsynaptic and not presynaptic site is the locus for molecular fingerprint of resilience. It is possible, that the palmitoylation of AMPA and NMDA receptor subunits along with GDI-1 might be differentially affected in resilient and anhedonic animals. Whether and how these processes are manifested within behavioral groups is a matter of further study.

Although our study provides valuable insights into the molecular and structural mechanisms underlying stress resilience, it is not without limitations. First, we cannot precisely determine which specific neurons were marked by DiI in our experiments, which suggests the need for further research to accurately identify the neuronal populations involved in the observed morphological changes. Additionally, we lacked an acute stress control group, meaning that some of the observed effects could potentially be attributed to acute stress responses rather than chronic stress alone. Future studies should address these limitations by incorporating type-specific neuronal labelling, acute stress controls, and more complex and unpredictable stress sequences to enhance the observations.

In conclusion, we demonstrated that stress-resilient behavior in adult animals is an actively regulated process accompanied by a set of unique functional, proteomic and morphological features in the brain. In particular, we have shown that the most robust synaptic alterations underlying stress resilience are associated with the structure of dendritic spines and postsynaptic intracellular signaling pathways in the hippocampus. At the cellular level, these differences might be triggered by S-PALM of synaptic proteins and translated into the regulation of synaptic receptors. However, further studies are required to indicate the chemical kinetics of these processes and their role in the behavioral stress response.

## 4 Materials and methods

### 4.1 Animals

Ten-week-old male C57BL/6J mice (Medical University of Bialystok, Poland) were individually housed under a reverse 12/12 h light/dark cycle (lights on at 8:00 p.m.) with food and water available *ad libitum*. Male 12-week-old CD1 mice (Janvier Labs, France) were used as resident intruders in the social defeat stress procedure and kept with the stressed C57BL6J mice in the same animal room. Male 12-week-old Wistar rats (Mossakowski Medical Research Institute, Polish Academy of Sciences, Warsaw, Poland) were used for predator stress. Male 12-week-old C57BL/6J mice (Mossakowski Medical Research Institute, Polish Academy of Sciences, Warsaw, Poland) were used for isolation of CA1 *Stratum Radiatum* and *Stratum Oriens* subregions of hippocampus. All animal procedures were performed according to the guidelines of the Polish Ethical Committee on Animal Research (permission no. 132/2016, 2011/2020, 203/2021, 204/2021).

### 4.2 Mouse model of stress resilience based on chronic unpredictable stress (CUS)

To evaluate depressive-like behavior in the mouse model, we used the chronic unpredictable stress paradigm (CUS) and behavioral evaluation as we described previously (Krzystyniak et al., 2019; Bijata et al., 2022a). We have described a highly reproducible protocol with all the relevant technical details previously (Bijata et al., 2022b). Briefly, before CUS, C57BL/6J mice were subjected to 2 weeks of room acclimatization, consisting of 1 week of handling. Mice were weighed and their baseline sucrose preference (SPT0) was measured before the CUS procedure. Then, based on their baseline parameters, mice were assigned to a control and stress group housed in two separate rooms. The 2-week CUS protocol consisted of 2 out of 3 different types of stressors chosen in a semirandom manner and applied daily during the dark phase under red light in the following sequence of restraint stress, tail suspension, and social defeat stress, with an intersession of at least 3 h (Table 1). During each light phase during the stress protocol, the mice were exposed to a rat. To stabilize glucocorticoid levels after the last exposure to a stressor, the mice were left undisturbed overnight before beginning the sucrose preference test. Thus, 16 h after the last stressor, the mice underwent a sucrose preference test (SPT1), resulting in the determination of sucrose preference 24 h after the last stressor, and thereafter, the body weight measurements and the forced swim test were performed. All mice were sacrificed 12–16 h after the behavioral evaluation (36–38 h after the last stressor). To correlate the molecular, functional, and structural readouts of excitatory synaptic plasticity in the hippocampus in relation to animal behavior, two independent CUS experiments were performed in which within each animal, one hippocampus was subjected to synaptoneurosome isolation (mass spectrometry analysis) or DiI labeling (dendritic spine imaging), and the second hippocampus was subjected to electrophysiology. The experimental design is outlined in Fig. 1A.

**Table 1:** **Chronic unpredictable stress stressors schedule.** SS – social defeat stress, RS - restraint stress, RAT - exposure to a predator (rat), TS - tail suspension stress.

	DARK PHASE (red light)		LIGHT PHASE
	∼12 p.m.	∼4 p.m.	∼8 p.m.
**DAY 1**			RAT
**DAY 2**	SS	RS	RAT
**DAY 3**	TSS	SS	RAT
**DAY 4**	RS	TSS	RAT
**DAY 5**	SS	RS	RAT
**DAY 6**	TSS	SS	RAT
**DAY 7**	RS	TSS	RAT
**DAY 8**	SS	RS	RAT
**DAY 9**	TSS	SS	RAT
**DAY 10**	RS	TSS	RAT
**DAY 11**	SS	RS	RAT
**DAY 12**	TSS	SS	RAT
**DAY 13**	RS	TSS	RAT
**DAY 14**	SS	RS (∼3 p.m.)	

**Restraint stress (RS):** The mice were placed inside a plastic tube (26 mm internal diameter) for 2 h during the dark phase.

**Tail suspension stress (TSS):** The mice were subjected to the tail suspension procedure by being hanged from the tails with adhesive tape for 40 min during the dark phase. To prevent the mice from climbing their tails, plastic cylinders (4 cm × 0.5 cm) were placed at the base of their tails.

**Social defeat stress (SS):** During each 30 min social defeat session performed in the dark phase, aggressive CD1 animals were placed in the home cages of C57BL/6J mice in the stress group. CD1 aggressive mice were selected as the CD1 mice that attacked C57BL/6J mice in less than 60 s without injuring them. During each session, the C57BL/6J mice exhibited signs of social defeat stress, such as a flight response, submissive posture, and audible vocalization. If the mice in the stress group did not display signs of social defeat stress, then the CD1 mouse was changed to another CD1 mouse. In rare cases of physical harm that occurred between pairs of mice, aggressive CD1 individuals were immediately removed from the cage of the C57BL/6J resident mice.

**Predator stress (RAT):** The mice were individually introduced into transparent, well-ventilated cylinders (15 cm × 8 cm) with food and bedding. The cylinders were then placed for 12 h (08:00 p.m.–08:00 a.m.) into a rat home cage that contained a rat during the light phase. For the rest of the day (08:00 a.m.–08:00 p.m.), the mice and rats were housed in their home cages in the same experimental room.

### 4.3 Behavioral tests

#### 4.3.1 Sucrose preference test (SPT)

Mice were given free-choice access to 1% sucrose solution and water that were provided in identical bottles for 8 h during the dark phase under a reverse light dark cycle. The percentage of sucrose preference was calculated as follows:

Sucrose Preference = [Weight _Sucrose_solution_ / (Weight _Sucrose_solution_ + Weight _Water_)] ∗ 100%

The consumption of water and sucrose solution was estimated simultaneously in the control and experimental groups by weighing the bottles. To eliminate possible bias from side preference, the positions of the bottles were changed after 4 h of the test. Twenty-four hours before the baseline sucrose preference test performed before the CUS procedure (SPT0), 2.5% sucrose solution was given to all animals for 2 h to prevent the possible effects of taste neophobia. The other conditions of the test were performed as previously described (Krzystyniak et al., 2019; Bijata et al., 2022a; Strekalova et al., 2004; Strekalova and Steinbusch, 2010). Sucrose preference after CUS (SPT1) values of <70.7% in mice in the stress group, measured 24 h after the last stressor, was the criterion for “anhedonia”, defined by the difference between the control and stressed groups >2 × SD. Anhedonic mice were previously shown to display depressive-like behavior (Strekalova et al., 2004; Strekalova and Steinbusch, 2010). None of the control animals exhibited <70.7% sucrose preference in SPT1. Stressed mice with sucrose preference >70.7% at the end of the CUS experiment were defined as resilient animals. The SPT evaluation before (SPT0) and after CUS (SPT1) is outlined in Fig. 1B. For further experiments, we selected mice that best represented their phenotype, meaning those with consistent forced swim test and SPT results.

#### 4.3.2 Forced swim test (FST)

Each mouse was placed into a cylindrical glass containers (20 cm × 40 cm) filled with warm water (∼27 °C) for a 6 min swimming session. The test was conducted under red light during the dark phase of the light/dark cycle after 1 h of room acclimatization where the behavioral test was performed. The sum of the floating time during the last 4 min was measured by visual scoring offline and defined as the time spent immobile.

All behavioral scorings were performed blind to the treatment groups.

### 4.4 Acute brain slice electrophysiology

Acute hippocampal brain slices were obtained from control, anhedonic, and resilient mice (N at least 5 mice/group) according to the protocol described previously (Wójtowicz et al., 2016). The hippocampi from one hemisphere were dissected and cut into 350 μm thick slices using a vibratome (VT1200S, Leica, Germany) in ice-cold buffer that contained 75 mM sucrose, 87 mM NaCl, 2.5 mM KCl, 1.25 mM NaH_2_PO_4_, 25 mM NaHCO_3_, 0.5 mM CaCl_2_, 10 mM MgSO_4_∗7H_2_O, and 20 mM glucose, pH 7.4. Slices were recovered in the same solution for 15 min (32 °C) and were subsequently stored in oxygenated (95% O_2_, 5% CO_2_) artificial cerebrospinal fluid (aCSF) that contained 125 mM NaCl, 25 mM NaHCO_3_, 2.6 mM KCl, 1.25 mM NaH_2_PO_4_, 2.0 mM CaCl_2_, and 20 mM glucose, pH 7.4. Recordings were made in aCSF after 2 h of slice recovery. Schaffer collateral pathway or commissural/associational-CA3 synapses were stimulated with a concentric bipolar electrode at CA1 or CA3 subregions, respectively (0.1 Hz, 0.3 ms, FHC, Bowdoin, ME USA). Compound AMPAR- and NMDAR-mediated fEPSPs were recorded with glass micropipettes that were filled with aCSF (1–3 M Ω resistance) in the *Stratum Radiatum* of the CA1 or CA3 subregion of the hippocampus as described previously^88,134^. NMDAR-mediated signals were isolated from compound fEPSPs with the AMPA/kainate receptor antagonist DNQX (20 μM) and L-type calcium channel blocker nifedipine (20 μM) in Mg^2+^-free solutions, as described previously (Wójtowicz et al., 2016). At the end of each recording, the NMDAR antagonist APV (50 μM) was used to confirm the origin of the recorded fEPSPs. All of the drugs were obtained from Sigma-Aldrich (Poland) and Tocris (UK). Input–output (I–O) relationships were built for fEPSPs amplitudes upon monotonically increasing the stimuli in the range of 0–300 μA (13 points, applied once at 0.1 Hz). Baseline stimulation was set at 0.1 Hz, and for baseline and paired-pulse stimulation protocols (interstimulus interval 25 ms), the stimulation strength was set to 40% of the maximum fEPSP amplitude. For better data visualization, the input–output curves shown in Fig. 4A were fitted with Equation:

$$y\left(x\right)=\text{an}\;\text{arctg}\;\frac{x}{b}\text{.}$$

Once the data were fitted, more complicated dependences, e.g., fEPSP amplitude vs. fiber volley amplitude, were recovered by combining the fit in the form of z(x) (with parameters a, b) with an inverse of the relation (1) in the form of x(y), with x = b'tg $\frac{y}{a^{\prime }}$, which led to z = a arctg $\frac{b^{\prime }}{b}\text{tg}\frac{y}{a^{\prime }}$ (Fig. 4B–C).

Due to the fact, that the data do not fulfill the assumptions of mixed design ANOVA test, and because of the complex design of the experiment, we obtained the p-value using a randomization approach by Monte Carlo methods, which do not rely on assumptions about the underlying data distribution and allow us to preserve the full complexity of the raw data without introducing bias from binning (Ruxton et al., 2013). We computed statistical significance of the differences (two-tailed tests) between the stimulation current, fiber volley amplitudes and fEPSPs, V(I), obtained from the electrophysiological experiments (Fig. 4A–C). The p-values were calculated as follows: In the first step, we quantified the differences between the measured $V^{A}\left(I_{i}\right)$ and $V^{B}\left(I_{i}\right)$ curves using the $L_{2\;}$ norm, defined as

$$L_{2}=\sqrt{\sum_{i=0}^{N}{\left(V^{A}\left(I_{i}\right)-V^{B}\left(I_{i}\right)\right)}^{2}}\text{,}$$

where $I_{i}$ is the stimulation current for the *i-*th measurement point and *N* is the total number of the measurement points. To compute the p values, we created the null-hypothesis ensemble, using the subject randomization and bootstrap techniques (Davison and Hinkley, 1997). The p value for the difference between two V(I) curves was calculated as

$$p_{value}=\frac{\sum_{j=1}^{B}F\left(L_{2\;j}\geq L_{2\;true}\right)}{B+1}\text{,}$$

where *F* is the indicator function that takes the value one when its argument is true and zero when it is false, $L_{2\;j}$ is the $L_{2\;}$ norm for the *j-*th element of the null-hypothesis ensemble, $L_{2\;true}$ is the actual value of $L_{2\;}$ for the analyzed V(I) curves, and *B* is the number of randomizations (we used *B* = 1000). The electrophysiology data were analyzed using AxoGraphX software as described previously (Wójtowicz et al., 2016).

### 4.5 Synaptoneurosomes

After euthanasia by cervical dislocation, the mice were decapitated, and hippocampi were removed and homogenized with a Dounce homogenizer in 3 mL of buffer A (5 mM HEPES (pH 7.4), 0.32 M sucrose, 0.2 mM ethylenediaminetetraacetic acid (EDTA), 50 mM N-ethylmaleimide (NEM), and protease inhibitor cocktail. Nuclei and cell debris were pelleted by 5 min centrifugation at 2500×*g*. The supernatant was then centrifuged at 12000×*g* for 5 min. The obtained pellet fraction was layered over a discontinuous Ficoll (Sigma-Aldrich) gradient (4%, 6%, and 13%) and centrifuged at 70000×*g* for 45 min. The synaptoneurosomal fraction was collected in buffer A and centrifuged at 20000×*g* for 20 min. The pellet corresponded to the synaptoneurosomes fraction. Purified synaptoneurosomes were obtained from the hippocampus collected from one hemisphere of control, anhedonic, resilient animals (N mice/group = 5) and subjected to mass spectrometry analysis as described previously (Zareba-Koziol et al., 2019). The obtained synaptoneurosomal fraction was visualized using electron microscopy and is presented in Fig. S3.

### 4.6 Mass spectrometry

The S-PALM or total protein peptide mixture (20 μL) was applied to the nanoACQUITY UPLC Trapping Column (Waters, 186003514) using water containing 0.1% formic acid as the mobile phase and transferred to the nanoACQUITY UPLC BEH C18 Column (75 μm inner diameter; 250 mm long, Waters, 186003545) using an acetonitrile gradient in the presence of 0.1% formic acid with a flow rate of 250 nL/min. The column outlet was directly coupled to the ion source of the Thermo Orbitrap Elite mass spectrometer (Thermo Electron Corp., San Jose, CA, USA) working in the regime of data-dependent MS to MS/MS switch. HCD fragmentation was used. All MS runs were separated by blank runs to reduce the carry-over of peptides from previous samples. The results of measurements were processed using Mascot-Distiller 2.7.1 software (MatrixScience, London, UK, on-site license). The Mascot search engine (version 2.7.1) was used to compare data against the UniProtKB/Swiss-Prot database (Swissprot, 2020_02; 16,905 sequences). The search parameters were set to the following: taxonomy (*Mus musculus*), variable modifications – cysteine carbamidomethylation or N-malemideidation, methionine oxidation, peptide tolerance (5 ppm), fragment mass tolerance (5 ppm). Enzyme specificity was set for trypsin with one missed or nonspecific cleavage permitted. The mass calibration and data filtering described above were also carried out. The lists of the peptide sequences (SPL) that were identified in all of the LC–MS/MS runs from synaptoneurosomal fractions were merged into one peptide list using MascotScan software (http://proteom.ibb.waw.pl/mscan/, accessed on April 9, 2021). The SPL consists of sequences of peptides with Mascot scores exceeding the threshold value corresponding to a 5% expectation value and FDR of 1% calculated by the Mascot procedure. For proteome quantitative analysis, peptide intensities were determined as the surface of the isotopic envelope of the tagged isotopic envelopes. Before the analysis, quantitative values were normalized with LOWESS as described previously (Zareba-Koziol et al., 2019).

### 4.7 Functional bioinformatics analysis of mass spectrometry data

For integrative analysis, we used ClueGO software to observe differential proteins involved in the GO terms. The input list of proteins for each GO analysis was distinguished on the basis of proteomic data analysis and Venn diagram analysis. The lists of proteins are grouped in Supplementary Tables 1–2 included in the Supplementary Materials. Proteins were analyzed with ClueGO v2.6.4/CluePedia v1.6.5 to obtain complete Gene Ontological terms (GO) from our datasets. ClueGO integrates GO terms and creates an organized GO/pathway term network. The statistical test used for the node enrichment was based on a right-sided hypergeometric option with a Benjamini–Hochberg correction and kappa score of 0.5. As a reference set for term enrichment calculations, we utilized genes from the *Mus musculus* genome (NCBI unique Gene identifiers). GO enrichment was conducted for different sets of proteins, and p values < 0.05 were considered to be significant. ClueGO results are grouped in Fig. 3.

### 4.8 DiI staining of brain slices and morphometric analysis of dendritic spines

To visualize changes in the structure of dendritic spines, 1,10-dioctadecyl-3,3,3,30-tetramethylindocarbocyanine perchlorate (DiI) staining was performed on one brain hemisphere fixed by incubation for 1 h in 1.5% paraformaldehyde. The hemispheres were sliced using a Leica vibratome. Slices (140 μm thick) containing hippocampal structures were allowed to recover for at least 1.5 h at room temperature. Random cell labeling was performed using 1.6 μm tungsten particles (Bio–Rad, Hercules, CA, USA) that were coated with a propelled lipophilic fluorescent dye (DiI; Invitrogen) delivered to the cells by gene gun (Bio–Rad) bombardment. Images of hippocampal neurons covered with dendritic spines were acquired under 561 nm fluorescent illumination using a confocal microscope Zeiss LSM800 (63 × objective, 1.4 NA) at a pixel resolution of 1024 × 1024 with a 2.4 × zoom, resulting in a 0.07 μm pixel size. The analysis of dendritic spine structure and calculation of changes in spine parameters were performed as described previously (Krzystyniak et al., 2019; Bijata et al., 2022a; Magnowska et al., 2016). The images that were acquired from the brain slices were processed using ImageJ software (National Institutes of Health, Bethesda, MD, USA) and analyzed semiautomatically using custom-written SpineMagick software (patent no. WO/2013/021001). The analyzed dendritic spines belonged to secondary and ternary dendrites of pyramidal or granule neurons to reduce possible differences in spine morphology caused by the location of spines on dendrites with different ranks. Additionally, to ensure that our analysis focused on the glutamatergic population and to exclude potential confounding effects of GABAergic spiny neurons, we applied a spine density cut-off of 30 spines/100 μm. This threshold was specifically designed to exclude interneurons with low spine densities from our morphological analysis. To quantify the changes in spine shape, we analyzed the relative changes in the spine length-to-head-width ratio (the scale-free parameter). The spine projected length was determined by measuring the curvilinear length along a fitted virtual skeleton of the spine, using maximal intensity projection on x-y plane. The reason for using the projection was insufficient microscope resolution towards z-axis, that did not allow the spine segmentation and measurements towards z-axis. Due to the projection, we record obviously shorter length for the spines with tortuosity in z, however the study of Hosokawa et al. (1995), shows that 2-dim distribution of the spine length can be corrected in order to reconstruct the three-dimensional distribution by convoluting the distribution with the function representing inverse stereological projection. Because in our analysis we compared length for different groups, any systematic changes in length would be revealed in statistic tests, regardless whether the convolution has been applied or not. Head width was defined as the diameter of the largest spine section while excluding the bottom part of the spine (1/3 of the spine length adjacent to the dendrite). Dendritic segments of 5 animals per group (70–93 cells/group) were morphologically analyzed resulting in CTR N_*spines*_ = 7 735, N_*dendrites*_ = 116; ANH N_*spines*_ = 7918, N_*dendrites*_ = 120; RES N_*spines*_ = 7026 N_*dendrites*_ = 107. To assess dendritic length for spine density analysis, we measured curvilinear length along the analyzed dendritic segment, which was obtained by fitting an n-order polynomial resulting in CTR_*dendritic length*_ = 23795.67 μm, ANH_*dendritic length*_ = 27179.45 μm, and _RES*dendritic length*_ = 21996.42 μm. The spine number was counted manually by a trained neurobiologist in ImageJ software. The statistical analysis was performed using nested analysis of variance (including the number of animals and the number of dendrite fragments in 1 field of view/spines analyzed per behavioral group. The distributions of spine parameters for spine density, length/head width, and projected area for the control, anhedonic and resilient groups are presented in Fig. S9.

### 4.9 Acyl-biotin exchange (ABE)

The acyl-biotin exchange (ABE) method was used to analyze protein palmitoylation in tissue samples from CA1, CA3 and DG subregions of the hippocampus of resilient, anhedonic and control animals. First, the samples were homogenized using Dounce homogenizer in a buffer containing 50 mM Tris HCl (pH 7.5), 150 mM NaCl, 1 mM EDTA, 4% SDS and 1% Triton X-100 (ABE buffer). The extracted proteins were reduced with 10 mM tris(2-carboxyethyl)phosphine (TCEP) and incubated with 50 mM N-ethylmaleimide (NEM) at 4°C for 16 h to block free thiol groups. Next, to remove the excess of unreacted NEM the proteins were precipitated with 96% ice-cold ethanol. The pellets were resuspended in the ABE buffer and the samples were treated with 400 μM thiol-reactive biotinylation reagent HPDP-biotin (N-[6-(biotinamido)hexyl]-3’-(2′-pyridyldithio)propionamide) and subsequently split into two equal parts. One part was treated with 1 M hydroxylamine to cleave thioester-linked palmitoyl moieties and expose freshly formed-thiols to HPDP-biotin; the other part was treated with Tris buffer pH 7.5 as a control which is used to identify unspecific binding of the HPDP-biotin. The samples were incubated in the dark for 1.5 h with constant agitation at room temperature. Finally, aliquots of the samples were subjected to SDS-PAGE and Western blotting for the analysis of the total palmitoylation (visualization of all biotinylated proteins) Stain-free total protein visualization was used as a loading control for total palmitoylation.

### 4.10 Western blot analysis

Protein samples were separated by SDS–PAGE and transferred to polyvinylidene difluoride membranes (Immobilon-P, Millipore). The membranes were then blocked with 10% nonfat milk in Tris-buffered saline with 0.1% Tween 20 (TBST). For the visualization of the total palmitoylation (biotinylated proteins after ABE) blocked membranes were incubated for 2 h in room temperature with Horseradish Peroxidase Avidin D (1:4000, A-2014, Vector Laboratories) diluted in 5% nonfat milk in TBST. After washing, the bands were detected using the SuperSignal West Femto Maximum Sensitivity Substrate (for CDC42 and 5-HT7R detection) or SuperSignal West Pico PLUS Chemiluminescent Substrate (for GAPDH detection) (Thermo Fisher Scientific).

### 4.11 Statistical analysis

Statistical analysis was performed using GraphPad Prism8 software (GraphPad, San Diego, CA, USA). One-way or two-way analysis of variance (one-way or two-way ANOVA) followed by post hoc tests was used for multiple comparisons to identify significant differences between experimental groups. In the case of unequal variances, the Welch correction was applied. If the data were not normally distributed, the Mann–Whitney test was used. Behavioral data were analyzed using one-way ANOVA followed by Bonferroni or Tukey post hoc tests. For the statistical analysis of structural plasticity of dendritic spines (density and morphology), we used nested-ANOVA statistics, including the number of animals and the number of dendrite fragments in one field of view for spines analyzed per behavioral group. The nested-ANOVA statistics were performed with R 4.1.1 language and environment for statistical computing (R Foundation for Statistical Computing, Vienna, Austria). The western blots were quantitatively analyzed by the sum of replicates (each data point on a replicate is divided by the sum of the values of all data points in that replicate) (Degasperi et al., 2014). A p value of <0.05 was considered statistically significant for all tests except for mass spectrometry-based palmitoylation analysis results in which <0.01 was used. The data are presented as the mean value ± SEM. All analyses were performed in a blinded manner. Information about the statistical test used is in the legend for each figure.

## CRediT authorship contribution statement

**E. Bączyńska:** Writing – review & editing, Writing – original draft, Methodology, Investigation, Data curation, Visualization, Conceptualization. **M. Zaręba-Kozioł:** Methodology, Investigation, Data curation. **B. Ruszczycki:** Software, Methodology, Data curation. **A. Krzystyniak:** Methodology, Data curation. **T. Wójtowicz:** Writing – review & editing, Visualization, Methodology, Investigation, Data curation. **K. Bijata:** Methodology, Investigation. **B. Pochwat:** Writing – original draft, Investigation. **M. Magnowska:** Investigation. **M. Roszkowska:** Investigation. **I. Figiel:** Investigation. **J. Masternak:** Investigation. **A. Pytyś:** Investigation, Data curation. **J. Dzwonek:** Investigation. **R. Worch:** Visualization, Investigation, Data curation. **K.H. Olszyński:** Investigation. **A.D. Wardak:** Investigation. **P. Szymczak:** Visualization, Data curation. **J. Labus:** Investigation. **K. Radwańska:** Methodology. **P. Jahołkowski:** Investigation. **A. Hogendorf:** Investigation. **E. Ponimaskin:** Supervision. **R.K. Filipkowski:** Supervision, Resources. **B. Szewczyk:** Supervision. **M. Bijata:** Writing – review & editing, Visualization, Supervision, Resources, Investigation, Funding acquisition, Data curation, Conceptualization. **J. Włodarczyk:** Writing – review & editing, Supervision, Resources, Funding acquisition, Conceptualization.

## Declaration of competing interest

The authors declare that they have no known competing financial interests or personal relationships that could have appeared to influence the work reported in this paper.

## Data Availability

All data supporting the findings of this study are available within the article or are available from the corresponding author upon reasonable request. The mass spectrometry proteomics data have been deposited to the ProteomeXchange Consortium via the PRIDE partner repository with the dataset identifier PXD026590.
