# Supplementary material for: Stress resilience is an active and multifactorial process manifested by structural, functional, and molecular changes in synapses
Source: Neurobiol Stress. 2024 Oct 22;33:100683. doi: 10.1016/j.ynstr.2024.100683 (PMC11543545; doi:10.1016/j.ynstr.2024.100683)
Supplement: Multimedia component 1 [file mmc1.docx]

**SUPPLEMENTAL FIGURES**


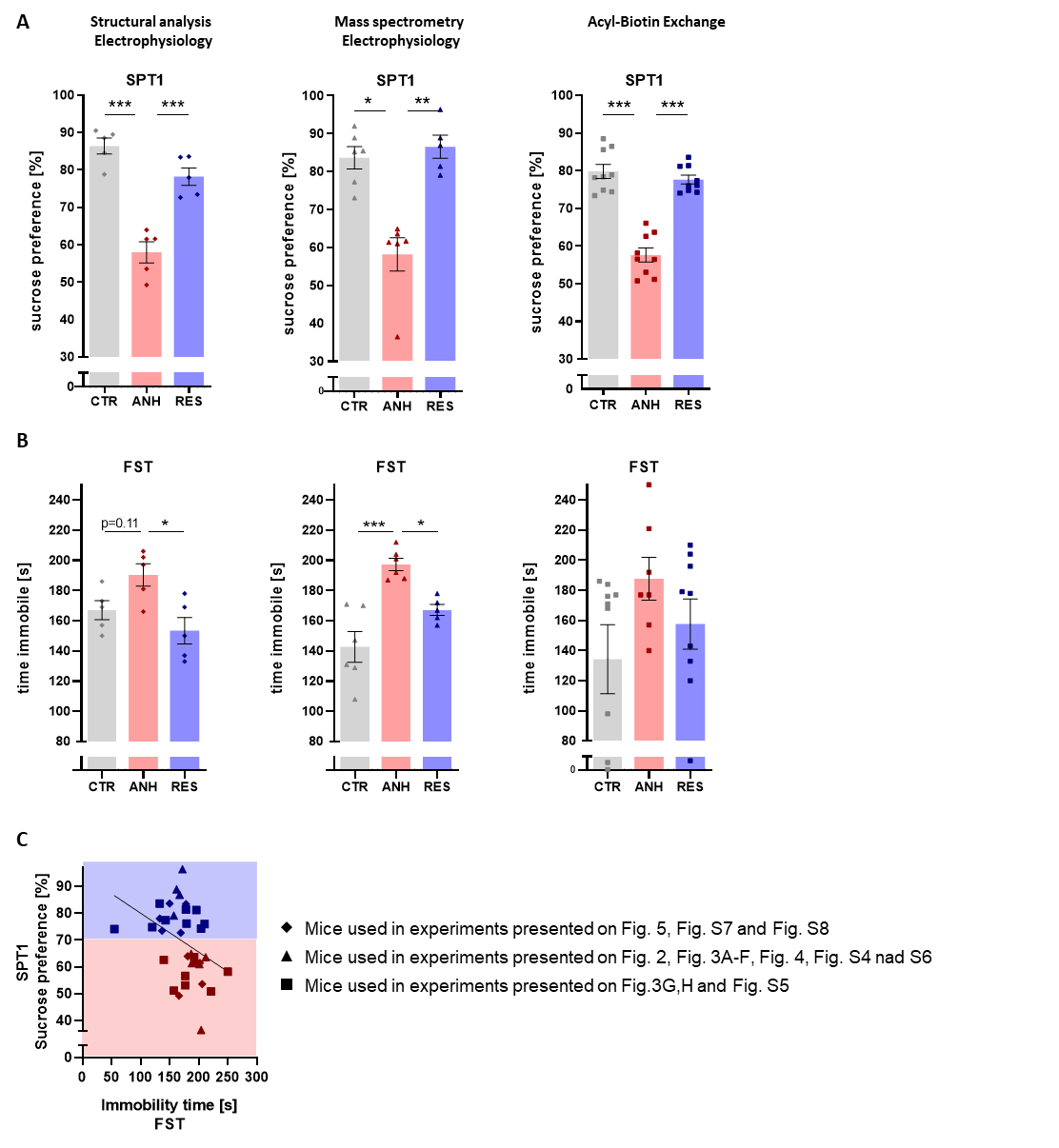


**Figure S1. Behavioral evaluation of depressive-like behavior of anhedonic (ANH) and resilient (RES) animals following chronic unpredictable stress (CUS) as well as control animals (CTR).** Behavioral parameters of animals used in the individual experiments described in the manuscript. **(A)** Sucrose Preference Test after CUS (SPT1). **(B)** Forced swim test after CUS (FST). The data are presented as the mean ± SEM. *p < 0.05; **p < 0.01; ***p < 0.001. (one-way ANOVA followed by Tukey's multiple comparisons test or (in case of SPT mass spectrometry and FST acyl-biotin exchange - Kruskal-Wallis test followed by Dunn's multiple comparisons test) **(C)** Correlation in the stress group between the behavioural outcomes in sucrose preference test (SPT1) and forced swimming test (FST). Pearson's correlation coefficient=-0.3771, R squared=0.1422; p=0.02; n = 37.


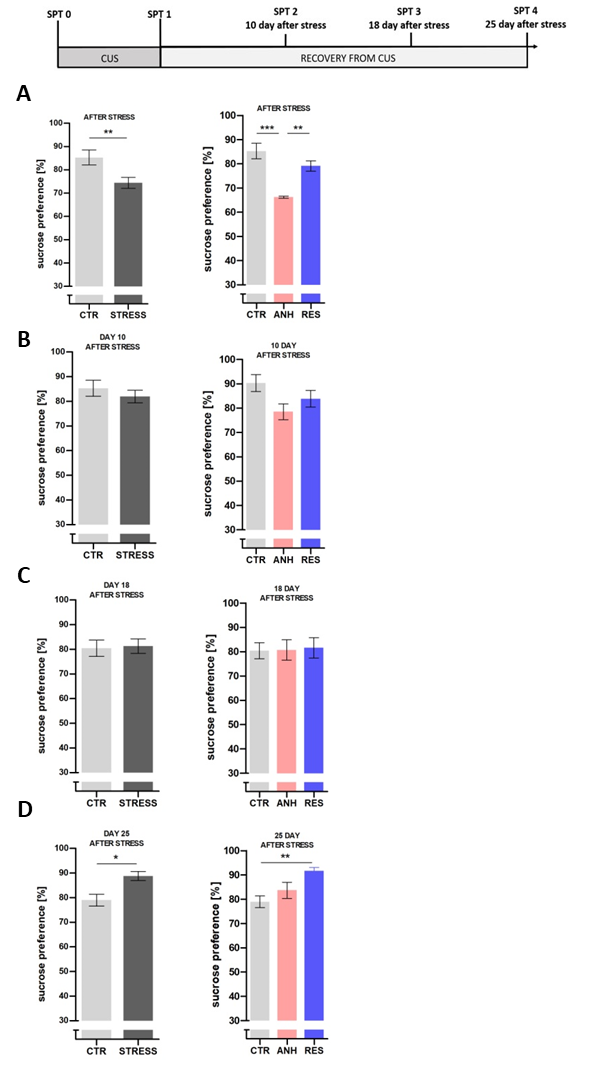


**Figure S2. Long term observation of recovery from chronic unpredictable stress (CUS).** Sucrose preference test performed: (A) after 2 weeks of CUS, (B) after 10 days from CUS, (C) after 18 days from CUS, (D) after 25 days from CUS. The data are presented as the mean value ± SEM (Mann-Whitney test with Dunn's multiple comparisons test). The results obtained at 10 and 18 days after chronic stress indicate that anhedonic animals recover after cessation of stress, while the resilient phenotype stabilizes. At 25 days after chronic stress, resilient animals showed an increase in sucrose preference, indicating possible hedonic behavior; however, to evaluate this phenomenon, further studies are needed, including more complex behavioral and molecular characterization after long-term recovery from chronic stress.


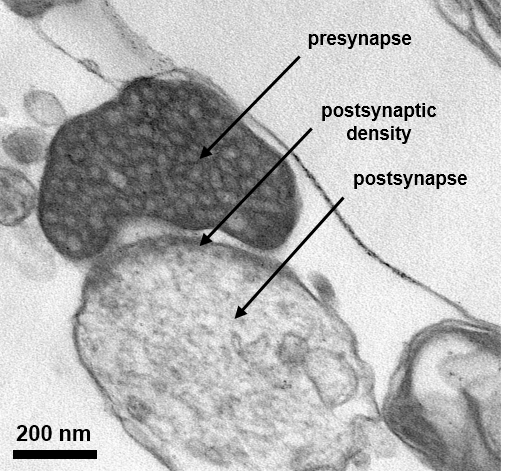


**Figure S3. Identification of presynaptic and postsynaptic parts in the synaptoneurosomal hippocampal fraction using electron microscopy.**

**Figure S4. Palmitoylation of selected cysteines differing between controls (CTR), anhedonic (ANH) and resilient (RES) mice.** The y-axis represents log2 signal intensity. Kruskal-Wallis test with Dunn’s multiple comparisons test. N_mice_=5 per group. Data are presented as the mean ± SEM. *p < 0.05; **p < 0.01, ***p < 0.001


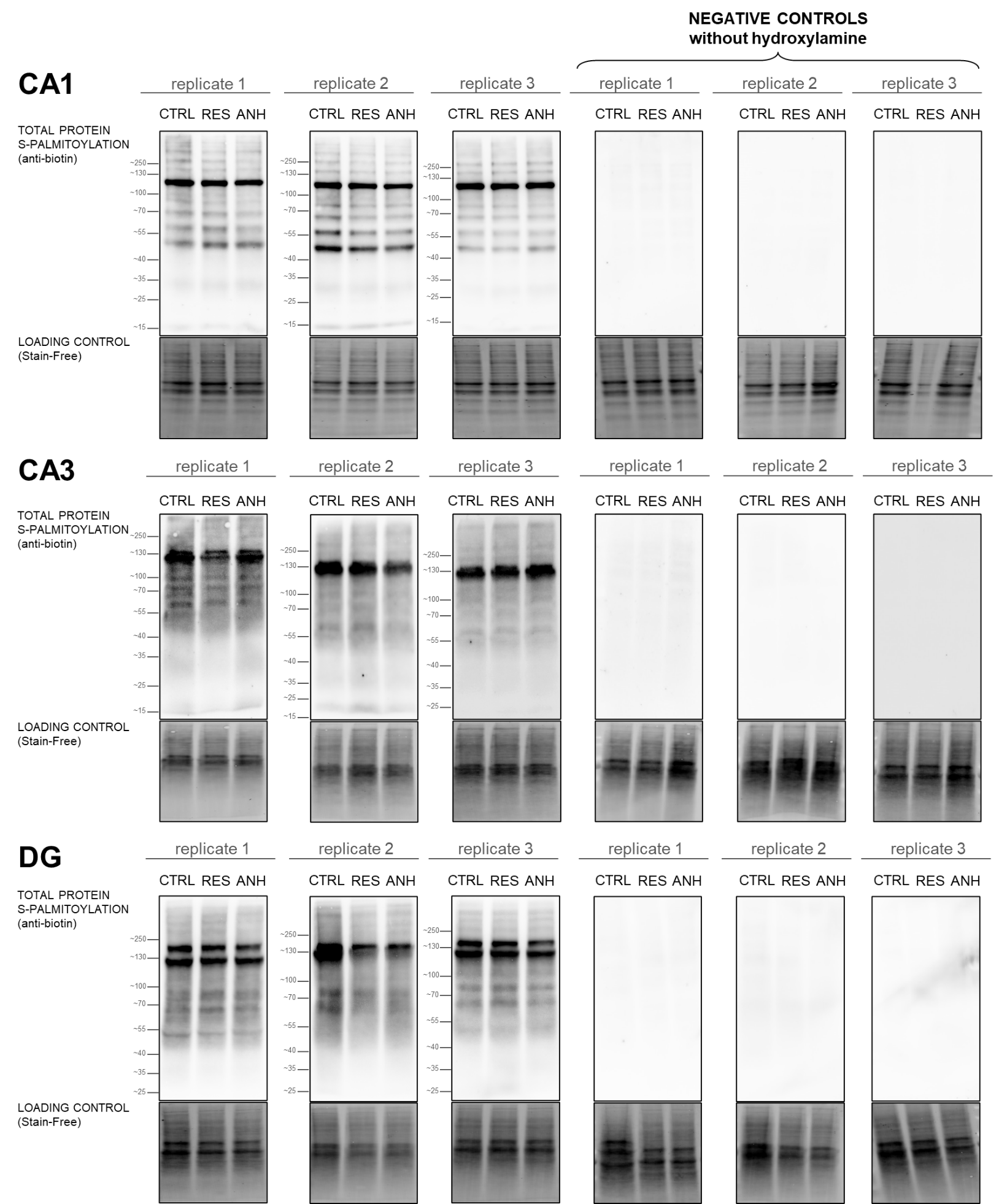


**Figure S5. Total palmitoylation of proteins in different subregions of the hippocampus (CA1, CA3, DG) of CTR, ANH, and RES animals.** The experiment was performed in triplicate, using pooled samples from three independent animals per each condition (n = 9). Total palmitoylation was visualized using an antibiotin antibody, while stain-free imaging was employed to visualize all proteins. Samples without added hydroxylamine served as negative controls to identify nonspecific binding of HPDP-biotin.


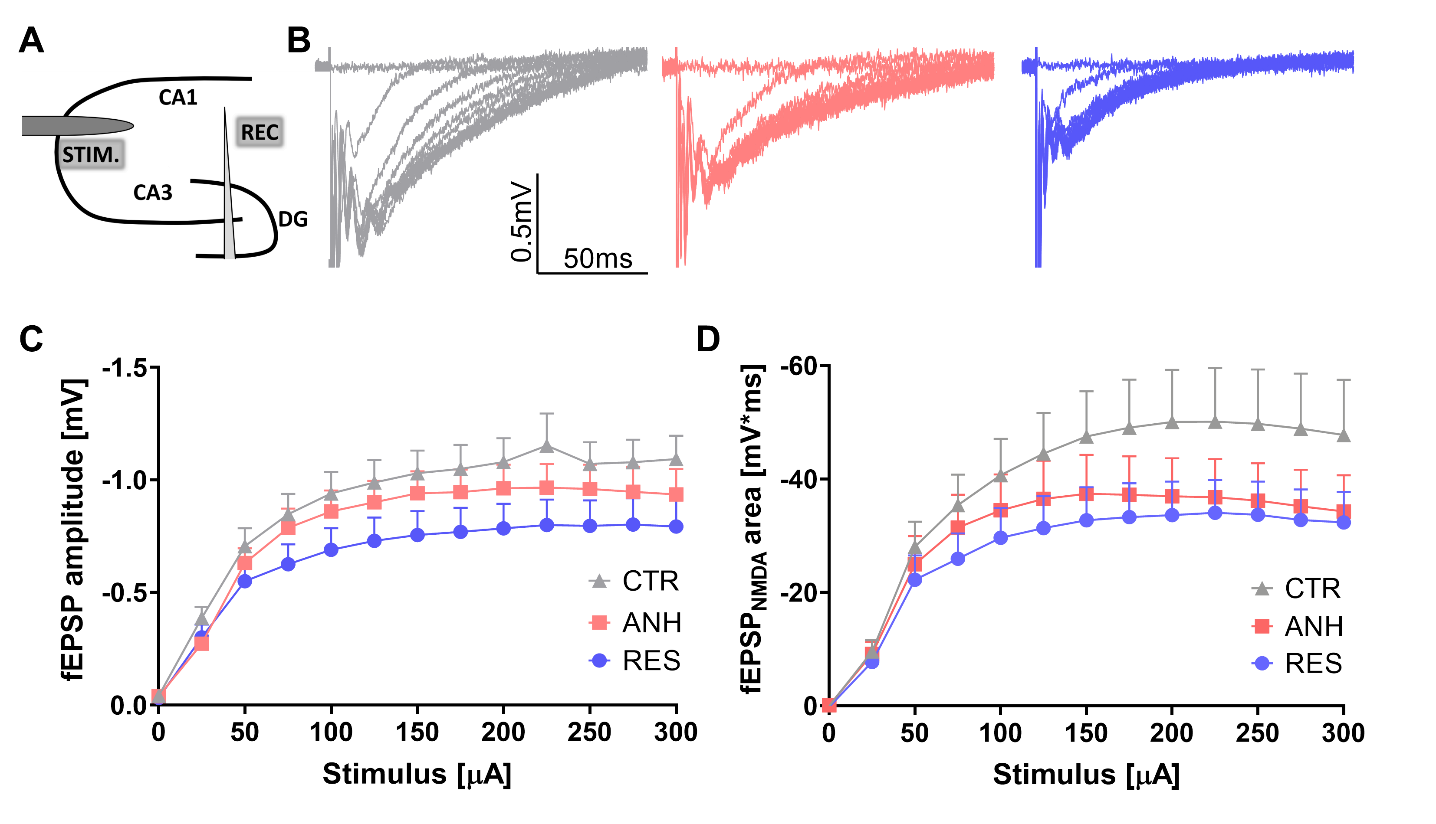


**Figure S6. (A)** Scheme of electrophysiological recordings **(B)** Exemplary compound fEPSPs evoked in the CA1 region of acute hippocampal slices in response to monotonically increased current stimuli in control (gray), anhedonic (red) and resilient group (blue). **(C-D)** Average input–output relationships for AMPAR-mediated **(C)** and NMDAR-mediated **(D)** fEPSPs. The average fEPSP amplitude and area did not differ between groups (CTR vs RES p=0.9, CTR vs ANH p=0.08, ANH vs RES p=0.16 for fEPSP amplitudes, and CTR vs RES p=0.8, CTR vs ANH p=0.37, ANH vs RES p=0.55 for fEPSP area, respectively, Monte Carlo simulations, n_slices_=15-20 (C) and n_slices_=12-16 (D), N_animals_=5-6 per group, for all comparisons). Data are presented as the mean ± SEM.

**
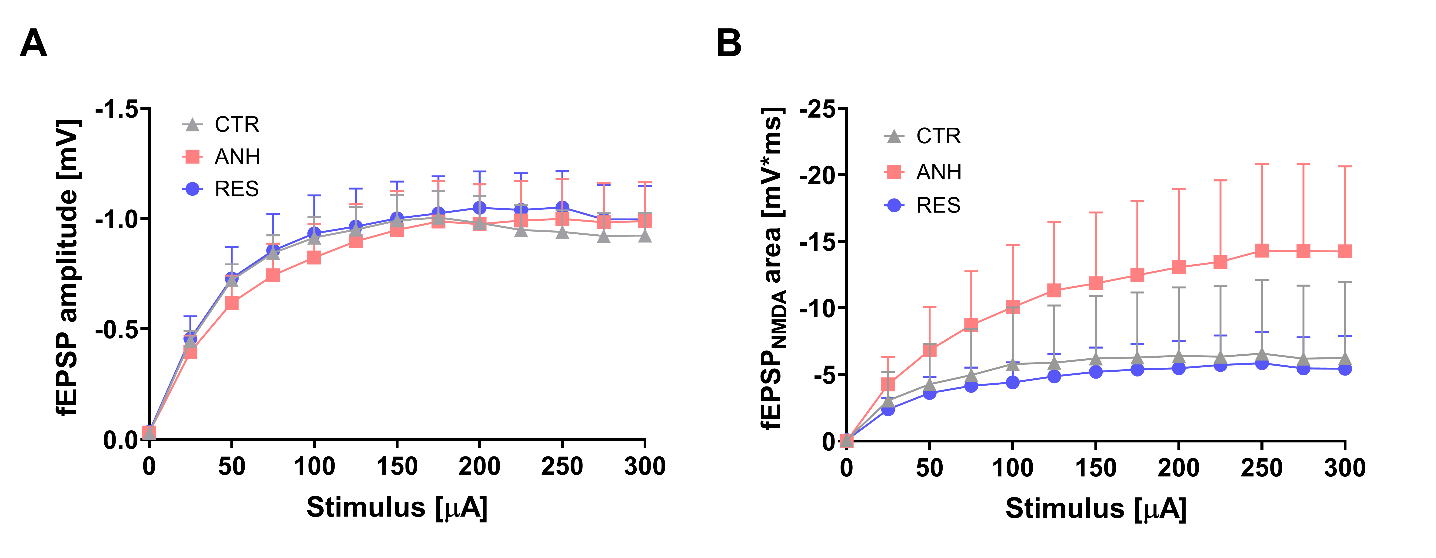
**

**Figure S7. Chronic stress differentially affects NMDAR-mediated neurotransmission in the CA3 region** **of the hippocampus.** **(A-B)** Average input–output relationships for AMPAR-mediated **(A)** and NMDAR-mediated **(B)** fEPSPs evoked in the CA3 region of acute hippocampal slices in response to monotonically increased current stimuli. **(A)** CUS did no results in any change in fEPSP amplitudes (n_slices_=6-8, N_animals_=4-6 per group, p>0.7 for all comparisons, Monte-Carlo simulations). **(B)** The area of the NMDAR-mediated component of fEPSPs was significantly upregulated in Anhedonic compared to Resilient group (p=0.031) while other groups did not differ significantly (n_slices_=3-5, N_animals_=3-6 per group, CTR vs RES p=0.87, CTR vs ANH p=0.085, Monte-Carlo simulations). Data are presented as the mean ± SEM.

**
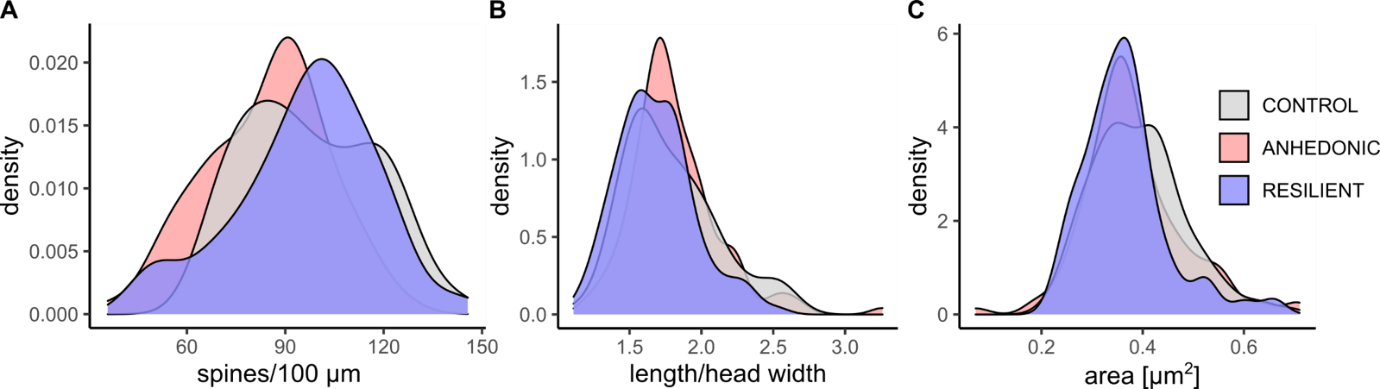
**

**
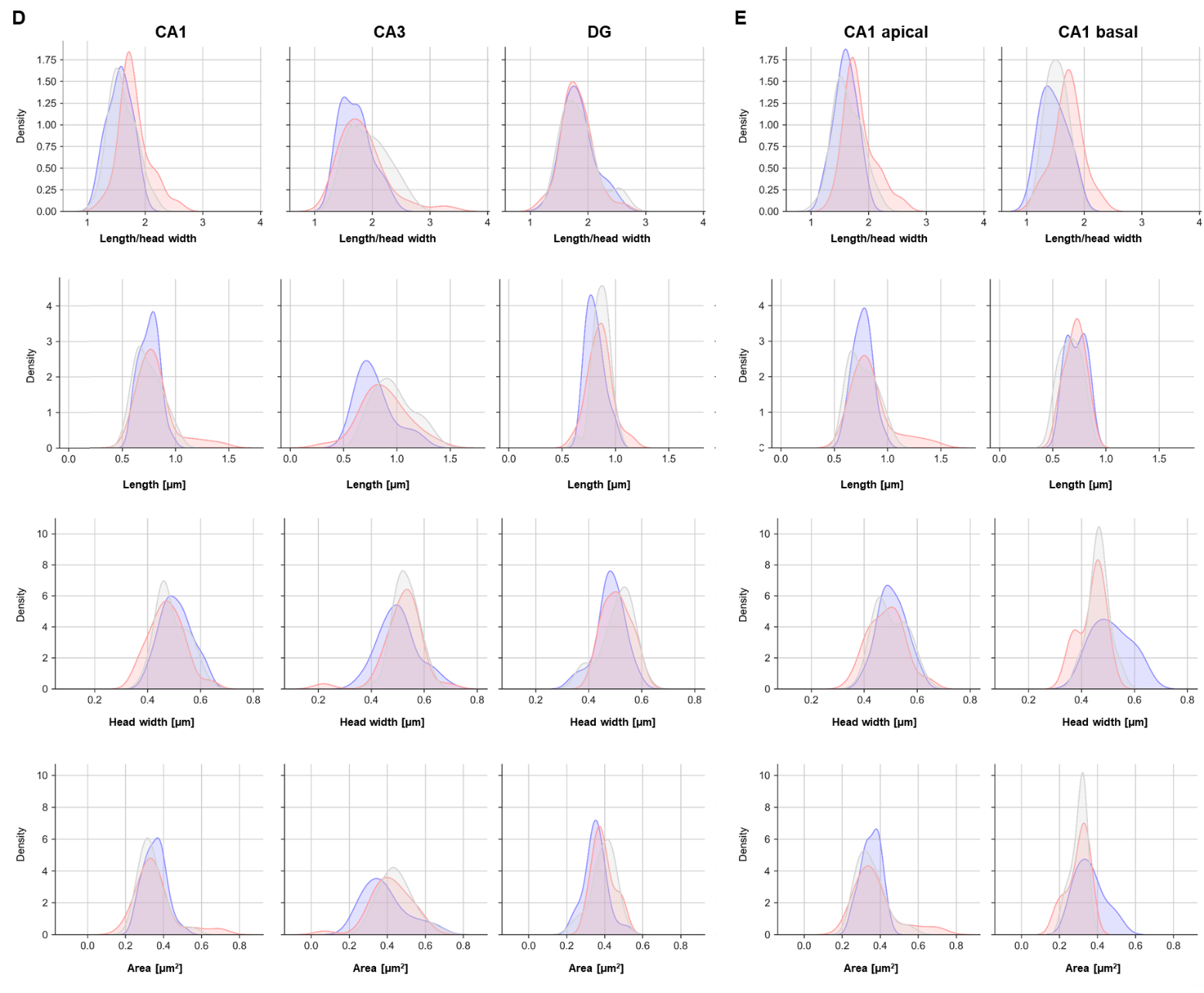
**

**Figure S8. Distributions of (A) spine density, (B) length/head width, and (C) area of dendritic spines in the hippocampus of CTR, ANH, RES animals.** **(D)** Distributions of length/head width, length, head width and area of dendritic spines in the CA1, CA3 and DG subregions of hippocampus of CTR, ANH, RES animals. **(E)** Distributions of length/head width, length, head width and area of dendritic spines localized in the apical and basal dendrites in the CA1 subregion of the hippocampi of CTR, ANH, RES animals. The distributions show that the density of probability integrated with the variable (labelled “density”) on the x-axis is normalized to unity.

**
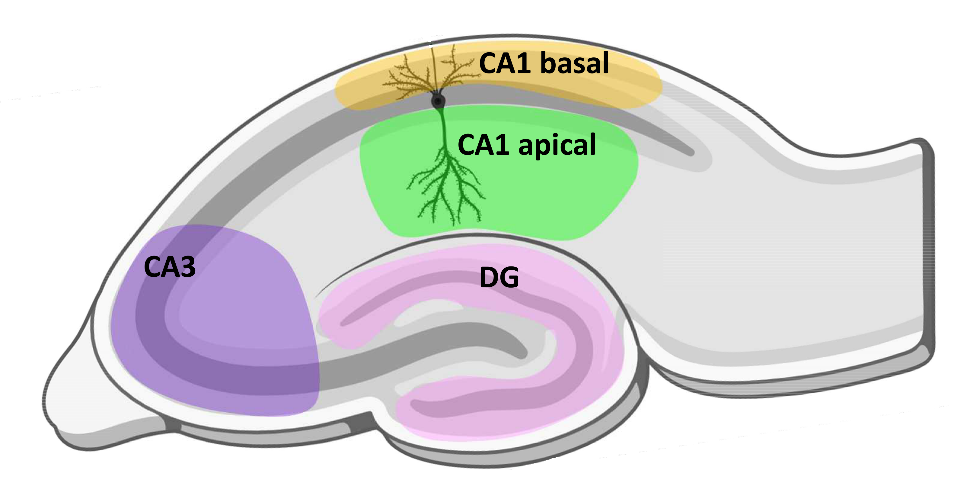
**

**Figure S9.** **Scheme illustrates the hippocampus, highlighting the specific territories of apical and basal dendrites in the CA1 and CA3 fields, as well as the dentate gyrus (DG).**

**Supplemental Table 1A. List of differential synaptic proteins between the control (CTR) and resilient (RES) mice.**

| **GENE NAME** | **UNIPORT ID** | **CTR/RES '+' = more in CTR '-' = more in RES** | **P-VALUE (CTR/RES) 0.05 = 1.33** | **ANNOTIATION** |
| --- | --- | --- | --- | --- |
| Usp9x | P70398 | 3.31 | 11.27 | Probable ubiquitin carboxyl-terminal hydrolase FAF-X |
| Fam213b | Q9DB60 | 0.69 | 6.90 | Prostamide/prostaglandin F synthase |
| Serpina3a | Q6P4P1 | 0.68 | 6.10 | Serine (or cysteine) peptidase inhibitor, clade A, member 3A |
| Nipsnap3b | Q9CQE1 | 0.68 | 1.73 | Nipsnap homolog 3B (*C. elegans*) |
| Odf3l2 | Q3TZ65 | 0.68 | 11.13 | Outer dense fiber of sperm tails 3-like 2 |
| Mtx2 | O88441 | 0.67 | 1.74 | Metaxin-2 |
| Pafah1b2 | Q61206 | 0.66 | 9.32 | Platelet-activating factor acetylhydrolase IB subunit beta |
| Atp5k | Q06185 | 0.62 | 6.68 | ATP synthase subunit E |
| Specc1 | Q5SXY1 | 0.61 | 7.57 | Cytospin-B |
| Ctnnb1 | Q02248 | 0.61 | 6.62 | Catenin beta-1 |
| Rdh13 | Q8CEE7 | 0.58 | 1.45 | Retinol dehydrogenase 13 |
| Cps1 | Q8C196 | 0.55 | 10.58 | Carbamoyl-phosphate synthase [ammonia] |
| Ttbk2 | Q3UVR3 | 0.53 | 7.78 | Tau-tubulin kinase 2 |
| Sec16a | E9QAT4 | 0.53 | 3.94 | Protein transport protein sec16 |
| Ddr1 | Q03146 | 0.53 | 6.42 | Epithelial discoidin domain-containing receptor 1 |
| Psma7 | Q9Z2U0 | 0.52 | 4.11 | Proteasome subunit alpha type-7 |
| Kiaa1524 | Q8BWY9 | 0.52 | 7.71 | Protein CIP2A |
| Sipa1l1 | Q8C0T5 | 0.52 | 6.46 | Signal-induced proliferation-associated 1-like protein 1 |
| Top2a | Q01320 | 0.52 | 3.76 | DNA topoisomerase 2-alpha |
| Ddx17 | Q501J6 | 0.52 | 4.85 | Probable ATP-dependent RNA helicase DDX17 |
| Celf1 | P28659 | 0.51 | 2.40 | CUGBP Elav-like family member 1 |
| Mlph | Q91V27 | 0.50 | 3.73 | Melanophilin |
| BC030499 | Q5SYL1 | 0.50 | 4.23 | Uncharacterized serine/threonine-protein kinase SgK494 |
| Tlx1 | P43345 | 0.50 | 6.27 | T-cell leukemia homeobox protein 1 |
| Rpl13 | P47963 | -0.50 | 9.77 | Ribosomal protein L13 |
| Nln | Q91YP2 | -0.50 | 3.71 | Neurolysin |
| Lmna | P48678 | -0.51 | 6.28 | Prelamin-A/C |
| Baiap2 | Q8BKX1 | -0.51 | 6.48 | Brain-specific angiogenesis inhibitor 1-associated protein 2 |
| Got2 | P05202 | -0.52 | 5.36 | Aspartate aminotransferase |
| Micu1 | Q8VCX5 | -0.52 | 9.39 | Calcium uptake protein 1 |
| Ext2 | P70428 | -0.52 | 7,07 | Exostosin-2 |
| Tomm22 | Q9CPQ3 | -0.52 | 3.42 | Mitochondrial import receptor subunit TOM22 homolog |
| Rab39b | Q8BHC1 | -0.53 | 6.41 | Ras-related protein Rab-39B |
| Ddias | Q6NZG4 | -0.53 | 7.15 | DNA damage-induced apoptosis suppressor protein |
| Rpl7a | P12970 | -0.55 | 5.33 | Ribosomal protein L7A |
| Phb | P67778 | -0.56 | 5.38 | Prohibitin |
| Epha4 | Q03137 | -0.57 | 7.69 | Ephrin type-A receptor 4 |
| Cdk17 | Q8K0D0 | -0.58 | 1.62 | Cyclin-dependent kinase 17 |
| Efr3b | Q6ZQ18 | -0.59 | 6.18 | Protein EFR3 homolog B |
| Icam5 | Q60625 | -0.61 | 9.00 | Intercellular adhesion molecule 5 |
| Podn | Q7TQ62 | -0.62 | 7.57 | Podocan |
| Dlg1 | Q811D0 | -0.62 | 9.13 | Disks large homolog 1 |
| Vps53 | Q8CCB4 | -0.65 | 7.73 | Vacuolar protein sorting-associated protein 53 homolog |
| Zdhhc5 | Q8VDZ4 | -0.83 | 8.36 | Palmitoyltransferase ZDHHC5 |

**Supplemental Table 1B. List of differential synaptic proteins between the control (CTR) and andehonic (ANH) mice.**

| **GENE NAME** | **UNIPORT ID** | **CTR/ANH ('+'=more in CTR,**  **'-'= more in ANH)** | **P-VALUE (CTR/ANH) 0.05 = 1.33** | **ANNOTIATION** |
| --- | --- | --- | --- | --- |
| Usp9x | P70398 | 3.50 | 11.28 | Probable ubiquitin carboxyl-terminal hydrolase FAF-X |
| Ubr2 | Q6WKZ8 | 1.24 | 1.95 | E3 ubiquitin-protein ligase UBR2 |
| Cox4i1 | P19783 | 1.14 | 1.97 | Cytochrome c oxidase subunit 4 isoform 1 |
| Ppid | Q9CR16 | 1.08 | 1.98 | Peptidyl-prolyl cis-trans isomerase D |
| Acot10 | Q32MW3 | 1.08 | 1.98 | Acyl-coenzyme A thioesterase 10 |
| Adsl | P54822 | 0.83 | 2.30 | Adenylosuccinate lyase |
| Ppp3ca | P63328 | 0.80 | 9.94 | Serine/threonine-protein phosphatase 2B catalytic subunit alpha isoform |
| Odf3l2 | Q3TZ65 | 0.61 | 10.88 | Outer dense fiber of sperm tails 3-like 2 |
| Bcl3 | Q9Z2F6 | 0.60 | 10.96 | B-cell lymphoma 3 protein homolog |
| Maob | Q8BW75 | 0.60 | 10.90 | Amine oxidase [flavin-containing] B |
| Slc22a21 | Q9WTN6 | 0.57 | 6.97 | Solute carrier family 22 member 21 |
| Fam83d | Q9D7I8 | 0.53 | 9.62 | Protein FAM83D |
| Mtm1 | Q9Z2C5 | 0.52 | 3.35 | Myotubularin |
| Tnfaip1 | O70479 | 0.52 | 10.67 | BTB/POZ domain-containing adapter for CUL3-mediated RhoA degradation protein 2 |
| Prkg2 | Q61410 | 0.51 | 2.96 | cGMP-dependent protein kinase 2 |
| Serpina3a | Q6P4P1 | 0.51 | 2.14 | Serine (or cysteine) peptidase inhibitor, clade A, member 3A |
| Maoa | Q64133 | 0.50 | 10.42 | Amine oxidase [flavin-containing] A |
| Klc3 | Q91W40 | 0.50 | 8.91 | Kinesin light chain 3 |
| Shank3 | Q4ACU6 | 0.50 | 8.75 | SH3 and multiple ankyrin repeat domains protein 3 |
| Hras | Q61411 | 0.50 | 6.95 | GTPase Hras |
| Wfs1 | P56695 | -0.50 | 4.62 | Wolframin |
| Tenm1 | Q9WTS4 | -0.51 | 3.44 | Teneurin-1 |
| Taf7l | Q9D3R9 | -0.51 | 10.40 | Transcription initiation factor TFIID subunit 7-like |
| Ext2 | P70428 | -0.52 | 12.01 | Exostosin-2 |
| Phb | P67778 | -0.52 | 11.97 | Prohibitin |
| Snx4 | Q91YJ2 | -0.52 | 3.68 | Sorting nexin-4 |
| Oxct1 | Q9D0K2 | -0.53 | 12.24 | Succinyl-CoA:3-ketoacid coenzyme A transferase 1 |
| Nln | Q91YP2 | -0.53 | 12.21 | Neurolysin |
| Epha4 | Q03137 | -0.53 | 6.97 | Ephrin type-A receptor 4 |
| Ddias | Q6NZG4 | -0.53 | 12.13 | DNA damage-induced apoptosis suppressor protein |
| Tagln3 | Q9R1Q8 | -0.54 | 12.32 | Transgelin-3 |
| Zdhhc13 | Q9CWU2 | -0.55 | 2.76 | Palmitoyltransferase ZDHHC13 |
| Efr3b | Q6ZQ18 | -0.56 | 12.38 | Protein EFR3 homolog B |
| Dlg1 | Q811D0 | -0.57 | 12.55 | Disks large homolog 1 |
| Gphn | Q8BUV3 | -0.60 | 12.64 | Gephyrin |
| Podn | Q7TQ62 | -0.61 | 12.76 | Podocan |
| Nrxn3 | Q6P9K9 | -0.63 | 1.34 | Neurexin-3 |
| Vps53 | Q8CCB4 | -0.65 | 13.01 | Vacuolar protein sorting-associated protein 53 homolog |
| Zdhhc5 | Q8VDZ4 | -0.80 | 9.94 | Palmitoyltransferase ZDHHC5 |

**Supplemental Table 1C. List of differential synaptic proteins between the anhedonic (ANH) and resilient (RES) mice.**

| **GENE NAME** | **UNIPROT ID** | **RES/ANH**  **('+'=more in RES,**  **'-'= more in ANH)** | **P-VALUE**  **(RES/ANH) 0.05 = 1.33** | **ANNOTATION** |
| --- | --- | --- | --- | --- |
| Ppp3ca | P63328 | 0.83 | 8.36 | Serine/threonine-protein phosphatase 2B catalytic subunit alpha isoform |
| Recql | Q9Z129 | -0.50 | 2.54 | ATP-dependent DNA helicase Q1 |
| Tenm1 | Q9WTS4 | -0.50 | 3.19 | Teneurin-1 |
| Fastkd1 | Q6DI86 | -0.51 | 9.54 | FAST kinase domain-containing protein 1 |
| Gcc2 | Q8CHG3 | -0.53 | 4.39 | GRIP and coiled-coil domain-containing protein 2 |
| Zdhhc13 | Q9CWU2 | -0.60 | 2.92 | Palmitoyltransferase ZDHHC13 |

**Supplemental Table 2A. List of synaptic proteins and palmitoylated cysteins with distinct palmitoylation profiles between the control (CTRL), resilient (RES) and anhedonic (ANH) mice.**

| **SEQUENCE** | **UNIPROT ID** | **NAME** | **CTRL** | **RES** | **ANH** |
| --- | --- | --- | --- | --- | --- |
| VETALEAC*SLPSSR | Q9Z1B3 | Plcb1 | **+** | **-** | **+** |
| VVLPSLAC*LR | Q9Z1B3 | Plcb1 | **+** | **+** | **-** |
| VFLNNLC*PRPEIDNIFSEFGAK | Q9Z1B3 | Plcb1 | **+** | **+** | **+** |
| SIKETIC*SQDER | Q9WUM4 | Coro1c | **+** | **+** | **+** |
| HVFGQAVKNDQC*YDDIR | Q9WUM4 | Coro1c | **+** | **+** | **+** |
| GLDVNKC*EIAR | Q9WUM4 | Coro1c | **+** | **+** | **+** |
| KC*EPIIMTVPR | Q9WUM4 | Coro1c | **+** | **+** | **-** |
| LKAAC*NLAR | Q9WUA3 | Pfkp | **+** | **+** | **+** |
| FVSDDSIC*VLGIC*KR | Q9WUA3 | Pfkp | **+** | **+** | **+** |
| GVFDC*R | Q9WUA3 | Pfkp | **+** | **+** | **+** |
| SNC*NVAVINVGAPAAGMNAAVR | Q9WUA3 | Pfkp | **+** | **+** | **+** |
| QC*QPTDTVC*ASVR | Q9WUC3 | Ly6h | **-** | **+** | **+** |
| QC*QPTDTVC*ASVR | Q9WUC3 | Ly6h | **-** | **+** | **+** |
| IIFVVGGPGSGKGTQC*EK | Q9R0Y5 | Ak1 | **+** | **+** | **+** |
| KVNAEGTVDTVFSEVC*TYLDSLK | Q9R0Y5 | Ak1 | **+** | **-** | **+** |
| AVIFC*LSADKK | Q9R0P5 | Dstn | **+** | **+** | **+** |
| KC*STPEEIK | Q9R0P5 | Dstn | **+** | **-** | **-** |
| TIC*VAYR | Q9R0K7 | Atp2b2 | **+** | **+** | **+** |
| IKETYGDTEAIC*R | Q9R0K7 | Atp2b2 | **+** | **+** | **+** |
| NESSHGGEFGC*TMEELR | Q9R0K7 | Atp2b2 | **-** | **+** | **+** |
| KVIEPMAC*DGLR | Q9R0K7 | Atp2b2 | **-** | **+** | **+** |
| IGAFGYMEC*SAK | Q9QUI0 | Rhoa | **+** | **+** | **+** |
| HFC*PNVPIILVGNKK | Q9QUI0 | Rhoa | **-** | **+** | **+** |
| NILVASPEC*VK | Q9QVP9 | Ptk2b | **+** | **+** | **+** |
| QLTSQDTKPTC*LAEFK | Q9QVP9 | Ptk2b | **+** | **-** | **+** |
| FFC*DPR | Q9ESW4 | Agk | **-** | **+** | **+** |
| KATVFLNPAAC*K | Q9ESW4 | Agk | **+** | **+** | **+** |
| ELSVQC*AGDWLPR | Q9DBE8 | Alg2 | **-** | **+** | **+** |
| VLFYC*HFPDLLLTQR | Q9DBE8 | Alg2 | **+** | **+** | **+** |
| HLVGVC*YTEEEAK | Q9D0M3 | Cyc1 | **+** | **+** | **+** |
| HGGEDYVFSLLTGYC*EPPTGVSLR | Q9D0M3 | Cyc1 | **-** | **+** | **+** |
| DSFPNFLAC*K | Q9CR61 | Ndufb7 | **-** | **+** | **+** |
| DYC*AHYLIR | Q9CR61 | Ndufb7 | **+** | **+** | **+** |
| EC*KIEFDDFEEC*LLR | Q99LY9 | Ndufs5 | **-** | **+** | **+** |
| C*HAFEKEWIEC*AHGIGGTR | Q99LY9 | Ndufs5 | **+** | **+** | **+** |
| DYEEIGPSIC*R | Q99JY9 | Actr3 | **-** | **+** | **+** |
| LGYAGNTEPQFIIPSC*IAIK | Q99JY9 | Actr3 | **+** | **+** | **+** |
| YSYVC*PDLVK | Q99JY9 | Actr3 | **+** | **+** | **+** |
| AAGSLLTDEC*R | Q922B1 | Macrod1 | **+** | **+** | **+** |
| SC*YLSSLDLLLEHR | Q922B1 | Macrod1 | **-** | **+** | **+** |
| NKVTC*LLYPHQVSAR | Q920I9 | Wdr7 | **-** | **+** | **+** |
| SLLVVC*SK | Q920I9 | Wdr7 | **+** | **+** | **+** |
| LPASC*LPASDSFR | Q920I9 | Wdr7 | **-** | **+** | **+** |
| NC*FHVAAVR | Q91ZP9 | Necab2 | **-** | **+** | **+** |
| HLQSPVC*K | Q91ZP9 | Necab2 | **+** | **-** | **-** |
| VC*LWIGR | Q91ZX7 | Lrp1 | **-** | **+** | **+** |
| LDGLC*IPLR | Q91ZX7 | Lrp1 | **+** | **+** | **+** |
| GVLFQPC*ER | Q91ZX7 | Lrp1 | **+** | **+** | **+** |
| ILQEDFTC*R | Q91ZX7 | Lrp1 | **+** | **+** | **+** |
| TAC*GVGEFR | Q91ZX7 | Lrp1 | **+** | **+** | **+** |
| C*LQGAC*VVNK | Q91ZX7 | Lrp1 | **+** | **+** | **+** |
| SGQQAC*EGVGSFLLYSVHEGIR | Q91ZX7 | Lrp1 | **+** | **+** | **+** |
| KFPFC*DGAHIK | Q91WS0 | Cisd1 | **+** | **+** | **+** |
| VVHAFDMEDLGDKAVYC*R | Q91WS0 | Cisd1 | **-** | **+** | **+** |
| SVIGGC*TQLLTHYYDDAR | Q91WC3 | Acsl6 | **-** | **+** | **+** |
| ALQPPC*NLLK | Q91WC3 | Acsl6 | **+** | **+** | **+** |
| LSVAGNC*R | Q91VD9 | Ndufs1 | **-** | **+** | **+** |
| FC*YHER | Q91VD9 | Ndufs1 | **+** | **+** | **+** |
| MC*LVEIEK | Q91VD9 | Ndufs1 | **+** | **+** | **+** |
| KGC*VITISGR | Q91V12 | Acot7 | **+** | **+** | **+** |
| C*VAALAR | Q91V12 | Acot7 | **-** | **+** | **+** |
| SVC*DYFFEAQER | Q8R2R9 | Ap3m2 | **-** | **-** | **+** |
| LLDDVSFHPC*VR | Q8R2R9 | Ap3m2 | **+** | **+** | **+** |
| SLAESIDDALNC*R | Q8R0S2 | Iqsec1 | **+** | **+** | **+** |
| AC*LDDSYASGEGLKR | Q8R0S2 | Iqsec1 | **-** | **+** | **+** |
| GAGPC*SPGLER | Q8R071 | Itpka | **-** | **+** | **+** |
| C*AAVAAAAAAGEPR | Q8R071 | Itpka | **+** | **+** | **+** |
| SC*ALSLLRR | Q8JZW5 | Sh2d5 | **+** | **+** | **+** |
| LNPTYEEQDC*GTEGRFPR | Q8JZW5 | Sh2d5 | **+** | **-** | **+** |
| LGNPYC*SPTLVR | Q8JZW5 | Sh2d5 | **+** | **+** | **+** |
| AC*LISR | Q8CHC4 | Synj1 | **-** | **+** | **+** |
| TSPC*QSPTVPEYSAPSLPIRPSRAPSR | Q8CHC4 | Synj1 | **+** | **+** | **+** |
| DGLTPLHC*AAR | Q8C8R3 | Ank2 | **+** | **+** | **+** |
| ATAVPDSLC*K | Q8C8R3 | Ank2 | **+** | **+** | **+** |
| GSC*GESQLPLVSSAFK | Q8C8R3 | Ank2 | **-** | **-** | **+** |
| QHAPVEIDEHPC*IEVR | Q8C8R3 | Ank2 | **+** | **+** | **+** |
| TPEDISTPPEGTKPC*LQTPVTSER | Q8C8R3 | Ank2 | **+** | **+** | **+** |
| SRDVEVLEGKPIYVDC*FGNLVPLTK | Q8C8R3 | Ank2 | **+** | **+** | **+** |
| LGYTPLIVAC*HYGNVK | Q8C8R3 | Ank2 | **-** | **-** | **+** |
| DVEVLEGKPIYVDC*FGNLVPLTK | Q8C8R3 | Ank2 | **+** | **-** | **+** |
| AGQVEVVRC*LLR | Q8C8R3 | Ank2 | **-** | **+** | **+** |
| NSGTVGAVALDC*R | Q8C0M9 | Asrgl1 | **+** | **+** | **+** |
| TPHC*FLTGHGAEK | Q8C0M9 | Asrgl1 | **-** | **-** | **+** |
| LGTVADC*GVPEAR | Q8BWF0 | Aldh5a1 | **+** | **+** | **+** |
| EVGEVLC*TDPLVSK | Q8BWF0 | Aldh5a1 | **+** | **+** | **+** |
| VAEQLEVGMVGVNEGLISSVEC*PFGGVK | Q8BWF0 | Aldh5a1 | **+** | **+** | **+** |
| NAGQTC*VC*SNRFLVQR | Q8BWF0 | Aldh5a1 | **+** | **-** | **-** |
| VGVNEGLISSVEC*PFGGVK | Q8BWF0 | Aldh5a1 | **+** | **-** | **-** |
| AFC*AENLEEK | Q8BWG8 | Arrb1 | **+** | **+** | **+** |
| AC*GVDYEVK | Q8BWG8 | Arrb1 | **+** | **+** | **+** |
| LSTASC*PTPK | Q8BUV3 | Gphn | **+** | **+** | **+** |
| VGVLTVSDSC*FR | Q8BUV3 | Gphn | **-** | **+** | **+** |
| AC*LLAAALGTTGER | Q8BNW9 | Kbtbd11 | **+** | **+** | **+** |
| ARPSDQVYC*YNPVTDSWSTVRPLR | Q8BNW9 | Kbtbd11 | **+** | **+** | **-** |
| LGNYVGAVQDC*ER | Q8BJU0 | Sgta | **+** | **+** | **+** |
| AIELNPANAVYFC*NR | Q8BJU0 | Sgta | **-** | **+** | **+** |
| NSFDC*FKK | Q8BH59 | Slc25a12 | **+** | **+** | **+** |
| AGQTTYSGVVDC*FR | Q8BH59 | Slc25a12 | **-** | **+** | **+** |
| DNILIEC*EAK | Q810U3 | Nfasc | **+** | **+** | **+** |
| ITNVSEEDSGEYFC*LASNK | Q810U3 | Nfasc | **-** | **-** | **+** |
| SGGRPEEYEGEYQC*FAR | Q810U3 | Nfasc | **+** | **+** | **+** |
| GTTVQLEC*R | Q810U3 | Nfasc | **+** | **+** | **+** |
| KEDQGIYTC*VATNILGK | Q810U3 | Nfasc | **-** | **-** | **+** |
| TRLDC*PFFGSPIPTLR | Q810U3 | Nfasc | **+** | **-** | **+** |
| TC*SIPIPTIIVKEPSTSSSGK | Q80Z38 | Shank2 | **+** | **+** | **+** |
| KPAGISNC*LPSSFLPPPESFDAVTDSGIEEVDSR | Q80Z38 | Shank2 | **-** | **+** | **+** |
| VSEC*SWPIR | Q80TZ3 | Dnajc6 | **+** | **+** | **+** |
| IYSTC*TDFER | Q80TZ3 | Dnajc6 | **+** | **+** | **+** |
| SGVAESAGLAC*SR | Q80TZ3 | Dnajc6 | **+** | **+** | **+** |
| LSDPAVC*GDR | Q80U23 | Snph | **-** | **+** | **+** |
| VGQAQVC*GSVLK | Q80U23 | Snph | **+** | **+** | **+** |
| VLC*LWR | Q80U28 | Madd | **+** | **+** | **+** |
| QTETGEGSVC*QR | Q80U28 | Madd | **+** | **+** | **+** |
| C*QVQGDPQPTVR | Q7TPD3 | Robo2 | **+** | **+** | **+** |
| TVTFPC*ETK | Q7TPD3 | Robo2 | **+** | **-** | **+** |
| LLPGEEC*VLDGLR | Q6ZPE2 | Sbf1 | **+** | **+** | **+** |
| QNRFPVVC*WR | Q6ZPE2 | Sbf1 | **-** | **-** | **+** |
| LGC*FDHAQR | Q6ZPJ3 | Ube2o | **+** | **+** | **+** |
| VQSC*PDPAVYGVVQSGDHVGR | Q6ZPJ3 | Ube2o | **-** | **+** | **+** |
| KNC*LVK | Q6PIC6 | Atp1a3 | **+** | **+** | **+** |
| LIIVEGC*QR | Q6PIC6 | Atp1a3 | **+** | **+** | **+** |
| STNC*VEGTAR | Q6PIC6 | Atp1a3 | **+** | **+** | **+** |
| AAVPDAVGKC*R | Q6PIC6 | Atp1a3 | **+** | **+** | **+** |
| ILDRC*ATILLQGK | Q6PIC6 | Atp1a3 | **+** | **+** | **+** |
| YNTDC*VQGLTHSK | Q6PIC6 | Atp1a3 | **+** | **+** | **+** |
| SPDC*THDNPLETR | Q6PIC6 | Atp1a3 | **+** | **+** | **+** |
| NLEAVETLGSTSTIC*SDK | Q6PIC6 | Atp1a3 | **+** | **+** | **+** |
| VLGFC*HYYLPEEQFPK | Q6PIC6 | Atp1a3 | **+** | **+** | **+** |
| DVAGDASESALLKC*IELSSGSVK | Q6PIC6 | Atp1a3 | **+** | **+** | **+** |
| IISAHGC*KVDNSSLTGESEPQTR | Q6PIC6 | Atp1a3 | **+** | **+** | **+** |
| AC*VIHGTDLKDFTSEQIDEILQNHTEIVFAR | Q6PIC6 | Atp1a3 | **+** | **+** | **+** |
| MSVEEVC*RK | Q6PIC6 | Atp1a3 | **+** | **+** | **+** |
| SSHTWVALSHIAGLC*NR | Q6PIC6 | Atp1a3 | **+** | **+** | **+** |
| C*ATILLQGK | Q6PIC6 | Atp1a3 | **+** | **+** | **+** |
| C*IELSC*GSVR | Q6PIE5 | Atp1a2 | **+** | **+** | **+** |
| ILDRC*STILVQGK | Q6PIE5 | Atp1a2 | **+** | **+** | **+** |
| DTAGDASESALLKC*IELSC*GSVR | Q6PIE5 | Atp1a2 | **+** | **+** | **+** |
| IISSHGC*KVDNSSLTGESEPQTR | Q6PIE5 | Atp1a2 | **+** | **+** | **+** |
| FQPHSGQEDLFSC*C*QR | Q6PDC8 | Mfsd4 | **-** | **+** | **+** |
| ALGVENPEC*YQR | Q6PDC8 | Mfsd4 | **+** | **+** | **+** |
| SLLDAC*EGRR | Q62261 | Sptbn1 | **-** | **+** | **+** |
| FATDGEGYKPC*DPQVIRDR | Q62261 | Sptbn1 | **+** | **-** | **-** |
| FIC*EQEHEK | Q61699 | Hsph1 | **+** | **+** | **+** |
| ELNNVC*EPVVTQPKPK | Q61699 | Hsph1 | **-** | **+** | **+** |
| GC*ALQC*AILSPAFK | Q61316 | Hspa4 | **+** | **+** | **+** |
| WNSPAEEGLSDC*EVFPK | Q61316 | Hspa4 | **+** | **+** | **+** |
| C*TPAC*VSFGPK | Q61316 | Hspa4 | **-** | **+** | **+** |
| SVMDATQIAGLNC*LR | Q61316 | Hspa4 | **-** | **+** | **+** |
| YQVDPDAC*FSAK | Q60932 | Vdac1 | **+** | **+** | **+** |
| EHINLGC*DVDFDIAGPSIR | Q60932 | Vdac1 | **+** | **-** | **+** |
| ALDLDSSC*KEAADGYQR | Q60864 | Stip1 | **+** | **+** | **+** |
| ALSAGNIDDALQC*YSEAIKLDPQNHVLYSNR | Q60864 | Stip1 | **-** | **+** | **+** |
| TEC*YGYALGDATR | Q60676 | Ppp5c | **-** | **-** | **+** |
| GVSC*QFGPDVTK | Q60676 | Ppp5c | **+** | **+** | **+** |
| QLVYEADGC*SPHGTLK | Q5DU25 | Iqsec2 | **-** | **+** | **+** |
| DVAQC*HLHHENPALGR | Q5DU25 | Iqsec2 | **+** | **+** | **+** |
| DGLTAVHC*ATR | Q4ACU6 | Shank3 | **+** | **+** | **-** |
| VLC*ALNHSLQDALNYGLFQPPSR | Q4ACU6 | Shank3 | **+** | **+** | **+** |
| GLDPNFHDPDSGEC*PLSLAAQLDNATDLLK | Q4ACU6 | Shank3 | **+** | **+** | **+** |
| ELNC*TELQELK | Q3UUG6 | Tbc1d24 | **+** | **+** | **+** |
| LIRDIPC*R | Q3UUG6 | Tbc1d24 | **-** | **-** | **+** |
| TEEFC*EIFR | Q3UHL1 | Camkv | **-** | **+** | **+** |
| AAATPEPAVAQPDSTALEGATGQAPPSSKGEEATGC*AQESQR | Q3UHL1 | Camkv | **-** | **+** | **+** |
| PFGC*VTLGDKK | Q3UHL1 | Camkv | **+** | **+** | **+** |
| GIYSC*IHGVAIEER | Q01097 | Grin2b | **+** | **+** | **+** |
| SYNNPPC*EENLFSDYISEVER | Q01097 | Grin2b | **-** | **-** | **+** |
| DSVSGGGPC*TNR | Q01097 | Grin2b | **-** | **-** | **+** |
| NLTNVDWEDRSGGNFC*R | Q01097 | Grin2b | **+** | **+** | **-** |
| AYSSFNPVMSDPNRPPC*R | Q00493 | Cpe | **+** | **-** | **+** |
| SGTAHEYSSC*PDDAIFQSLAR | Q00493 | Cpe | **+** | **+** | **+** |
| INC*PVYITK | P97427 | Crmp1 | **-** | **+** | **+** |
| STVEYNIFEGMEC*HGSPLVVISQGK | P97427 | Crmp1 | **+** | **+** | **+** |
| GC*DLVPR | P83093 | Stim2 | **-** | **+** | **+** |
| GSPEC*VGLTETK | P83093 | Stim2 | **+** | **+** | **+** |
| EAVAIC*K | P68404 | Prkcb | **-** | **-** | **+** |
| C*SLNPEWNETFR | P68404 | Prkcb | **+** | **+** | **+** |
| NVVTIFSAPNYC*YR | P63330 | Ppp2ca | **+** | **+** | **+** |
| LQEVPHEGPMC*DLLWSDPDDRGGWGISPR | P63330 | Ppp2ca | **-** | **+** | **+** |
| LWDFQGFEC*IR | P63005 | Pafah1b1 | **-** | **+** | **+** |
| MVRPNQDGTLIASC*SNDQTVR | P63005 | Pafah1b1 | **+** | **+** | **+** |
| SGQC*VQAFETHESDVNSVR | P62881 | Gnb5 | **+** | **+** | **+** |
| YYPSGDAFASGSDDATC*R | P62881 | Gnb5 | **-** | **+** | **+** |
| VSPDGTAFC*SGSWDHTLR | P62881 | Gnb5 | **+** | **+** | **+** |
| LFVSGAC*DASAK | P62874 | Gnb1 | **+** | **+** | **+** |
| ELAGHTGYLSC*C*R | P62874 | Gnb1 | **+** | **+** | **+** |
| ELAGHTGYLSC*C*R | P62874 | Gnb1 | **+** | **+** | **+** |
| AC*ADATLSQITNNIDPVGR | P62874 | Gnb1 | **+** | **+** | **+** |
| LLLAGYDDFNC*NVWDALKADR | P62874 | Gnb1 | **+** | **+** | **+** |
| ELAGHTGYLSC*C*R | P62874 | Gnb1 | **+** | **+** | **+** |
| ADQELMTYSHDNIIC*GITSVSFSK | P62874 | Gnb1 | **+** | **-** | **+** |
| NKGVVLGGC*GDK | P61922 | Abat | **+** | **+** | **+** |
| C*LEEVEDLIVK | P61922 | Abat | **+** | **+** | **-** |
| KVVVC*DNGTGFVK | P61161 | Actr2 | **+** | **-** | **-** |
| LC*YVGYNIEQEQK | P61161 | Actr2 | **+** | **+** | **+** |
| LAC*SEAPTDVSK | P58871 | Tnks1bp1 | **+** | **+** | **+** |
| LC*GAPSEVR | P58871 | Tnks1bp1 | **+** | **+** | **-** |
| RAELSGNC*SPLLIQPR | P59281 | Arhgap39 | **-** | **+** | **+** |
| NRKPSLC*QVPSTSSTDGAGGLLGEQPLTEER | P59281 | Arhgap39 | **+** | **+** | **+** |
| EFKGLGDC*LVK | P51881 | Slc25a5 | **-** | **-** | **+** |
| SLC*FVYPLDFAR | P51881 | Slc25a5 | **+** | **+** | **+** |
| KGTDIMYTGTLDC*WR | P51881 | Slc25a5 | **+** | **+** | **+** |
| GIFPVLC*K | P52480 | Pkm | **+** | **+** | **+** |
| NTGIIC*TIGPASR | P52480 | Pkm | **-** | **+** | **+** |
| TDDYLDQPC*LETINR | P50396 | Gdi1 | **-** | **-** | **+** |
| NTNDANSC*QIIIPQNQVNR | P50396 | Gdi1 | **+** | **+** | **+** |
| QLIC*DPSYIPDR | P50396 | Gdi1 | **+** | **+** | **+** |
| HLTEYFTFKQEC*K | P48453 | Ppp3cb | **+** | **+** | **+** |
| IC*SFEEAKGLDR | P48453 | Ppp3cb | **+** | **+** | **-** |
| TPC*AETPAEPVDWAFAQR | P33173 | Kif1a | **+** | **+** | **+** |
| TPQPC*SRPASPEPELLPELDSK | P33173 | Kif1a | **-** | **-** | **-** |
| LQEAC*KDILLFK | P31324 | Prkar2b | **-** | **+** | **+** |
| RASVC*AEAYNPDEEEDDAESR | P31324 | Prkar2b | **+** | **+** | **+** |
| LC*TGHEYAAK | P28652 | Camk2b | **+** | **+** | **+** |
| ITAHEALKHPWVC*QR | P28652 | Camk2b | **+** | **+** | **+** |
| WQNVHFHC*SGAPVAPLQ | P28652 | Camk2b | **+** | **+** | **+** |
| QETVEC*LK | P28652 | Camk2b | **-** | **+** | **+** |
| C*VKLC*TGHEYAAK | P28652 | Camk2b | **+** | **+** | **+** |
| GFC*LIPQQSINEAIR | P23818 | Gria1 | **-** | **+** | **+** |
| QGC*DISPR | P23818 | Gria1 | **-** | **+** | **+** |
| RGNAGDC*LANPAVPWGQGIDIQR | P23818 | Gria1 | **+** | **+** | **+** |
| TTQIGC*LLR | P17427 | Ap2a2 | **+** | **+** | **+** |
| LC*ELLSEQF | P17427 | Ap2a2 | **+** | **+** | **+** |
| TVFEALQAPAC*HENLVK | P17427 | Ap2a2 | **-** | **+** | **+** |
| SC*NC*LLLK | P17182 | Eno1 | **-** | **+** | **+** |
| VNQIGSVTESLQAC*K | P17182 | Eno1 | **+** | **+** | **+** |
| SGETEDTFIADLVVGLC*TGQIK | P17182 | Eno1 | **+** | **+** | **+** |
| VPTPNVSVVDLTC*R | P16858 | Gapdh | **-** | **+** | **+** |
| IVSNASC*TTNC*LAPLAK | P16858 | Gapdh | **+** | **+** | **+** |
| NC*LVGENLLVK | P15209 | Ntrk2 | **-** | **+** | **+** |
| IWC*TEPSPGIVAFPR | P15209 | Ntrk2 | **+** | **+** | **+** |
| SSPDTQDLYC*LNESSK | P15209 | Ntrk2 | **-** | **+** | **+** |
| VSLNC*R | P12960 | Cntn1 | **+** | **+** | **-** |
| VIIEC*KPK | P12960 | Cntn1 | **+** | **+** | **+** |
| FFLC*QVAGDAK | P13595 | Ncam1 | **+** | **+** | **+** |
| NVDKNDEAEYVC*IAENK | P13595 | Ncam1 | **-** | **+** | **+** |
| LDC*QVQGRPQPEITWR | P11627 | L1cam | **-** | **+** | **+** |
| TLQLLNVGEEDDGEYTC*LAENSLGSAR | P11627 | L1cam | **+** | **+** | **+** |
| TITC*TR | P11798 | Camk2a | **+** | **+** | **+** |
| QETVD**C***LKK | P11798 | Camk2a | **+** | **+** | **+** |
| STVASC*MHR | P11798 | Camk2a | **+** | **+** | **+** |
| C*VKVLAGQEYAAK | P11798 | Camk2a | **-** | **+** | **+** |
| MC*DPGMTAFEPEALGNLVEGLDFHR | P11798 | Camk2a | **-** | **+** | **+** |
| C*ASFFR | O35874 | Slc1a4 | **-** | **+** | **+** |
| C*IEENNGVDKR | O35874 | Slc1a4 | **+** | **-** | **-** |
| AFFPAC*YGGDGR | O35633 | Slc32a1 | **-** | **+** | **+** |
| SEGEPC*GDEGAEAPVEGDIHYQR | O35633 | Slc32a1 | **+** | **+** | **+** |
| LGVSPGGDAGTC*PPVGR | B9EJA2 | Cttnbp2 | **+** | **+** | **+** |
| SVAC*QTDVVTESTDPVK | B9EJA2 | Cttnbp2 | **-** | **-** | **+** |
| FC*VYTR | A2ALU4 | Shroom2 | **-** | **-** | **+** |
| TLSTSEQC*YSR | A2ALU4 | Shroom2 | **+** | **+** | **+** |

**Supplemental Table 2B. List of synaptic proteins with distinct palmitoylation profiles between the resilient (RES) and anhedonic (ANH) mice.**

| **GENE NAME** | **UNIPROT ID** | **STRING ID** |
| --- | --- | --- |
| Abat | P61922 | 10090.ENSMUSP00000063548 |
| Acot10 | Q32MW3 | 10090.ENSMUSP00000051333 |
| Actr3b | Q641P0 | 10090.ENSMUSP00000085578 |
| Ak1 | Q9R0Y5 | 10090.ENSMUSP00000068479 |
| Ank2 | Q8C8R3 | 10090.ENSMUSP00000043765 |
| Ap3d1 | O54774 | 10090.ENSMUSP00000020420 |
| Ap3m2 | Q8R2R9 | 10090.ENSMUSP00000128446 |
| Apc | Q61315 | 10090.ENSMUSP00000078337 |
| Asrgl1 | Q8C0M9 | 10090.ENSMUSP00000051709 |
| Atp11a | P98197 | 10090.ENSMUSP00000088779 |
| Bdh1 | Q80XN0 | 10090.ENSMUSP00000110882 |
| Cmtm4 | Q8CJ61 | 10090.ENSMUSP00000137332 |
| Cntn1 | P12960 | 10090.ENSMUSP00000000109 |
| Comtd1 | Q8BIG7 | 10090.ENSMUSP00000119330 |
| Coro1c | Q9WUM4 | 10090.ENSMUSP00000004646 |
| Cpe | Q00493 | 10090.ENSMUSP00000048555 |
| Cpt1c | Q8BGD5 | 10090.ENSMUSP00000069539 |
| Crtc1 | Q68ED7 | 10090.ENSMUSP00000075916 |
| Cttnbp2 | B9EJA2 | 10090.ENSMUSP00000088089 |
| Daglb | Q91WC9 | 10090.ENSMUSP00000043088 |
| Dars2 | Q8BIP0 | 10090.ENSMUSP00000041851 |
| Dhx9 | O70133 | 10090.ENSMUSP00000038135 |
| Dmxl2 | Q8BPN8 | 10090.ENSMUSP00000113705 |
| Eno3 | P21550 | 10090.ENSMUSP00000072620 |
| Epha4 | Q03137 | 10090.ENSMUSP00000027451 |
| Erc1 | Q99MI1 | 10090.ENSMUSP00000078534 |
| Fam19a2 | Q7TPG7 | 10090.ENSMUSP00000050199 |
| Frrs1l | B1AXV0 | 10090.ENSMUSP00000052507 |
| Gclc | P97494 | 10090.ENSMUSP00000034905 |
| Gdi1 | P50396 | 10090.ENSMUSP00000015435 |
| Gh | P06880 | 10090.ENSMUSP00000099360 |
| Gnaz | O70443 | 10090.ENSMUSP00000036087 |
| Gnb1 | P62874 | 10090.ENSMUSP00000030940 |
| Grin2b | Q01097 | 10090.ENSMUSP00000062284 |
| Htt | P42859 | 10090.ENSMUSP00000078945 |
| Idh2 | P54071 | 10090.ENSMUSP00000103007 |
| Kbtbd11 | Q8BNW9 | 10090.ENSMUSP00000068321 |
| Kcna2 | P63141 | 10090.ENSMUSP00000041702 |
| Kif1a | P33173 | 10090.ENSMUSP00000108582 |
| Lap3 | Q9CPY7 | 10090.ENSMUSP00000040222 |
| Lrp4 | Q8VI56 | 10090.ENSMUSP00000028689 |
| Mcu | Q3UMR5 | 10090.ENSMUSP00000020312 |
| Mpp6 | Q9JLB0 | 10090.ENSMUSP00000125880 |
| Mtx1 | P47802 | 10090.ENSMUSP00000073261 |
| Naaa | Q9D7V9 | 10090.ENSMUSP00000108726 |
| Nck2 | O55033 | 10090.ENSMUSP00000083611 |
| Nckap1 | P28660 | 10090.ENSMUSP00000028386 |
| Nfasc | Q810U3 | 10090.ENSMUSP00000092148 |
| Ntm | Q99PJ0 | 10090.ENSMUSP00000074578 |
| Oxr1 | Q4KMM3 | 10090.ENSMUSP00000105926 |
| Pdha1 | P35486 | 10090.ENSMUSP00000033662 |
| Phyhipl | Q8BGT8 | 10090.ENSMUSP00000045807 |
| Plcb1 | Q9Z1B3 | 10090.ENSMUSP00000105743 |
| Ppme1 | Q8BVQ5 | 10090.ENSMUSP00000032963 |
| Ppp1r1b | Q60829 | 10090.ENSMUSP00000077760 |
| Ppp2r5e | Q61151 | 10090.ENSMUSP00000021447 |
| Ppp3cb | P48453 | 10090.ENSMUSP00000125722 |
| Ppp5c | Q60676 | 10090.ENSMUSP00000003183 |
| Prkcb | P68404 | 10090.ENSMUSP00000070019 |
| Ptk2b | Q9QVP9 | 10090.ENSMUSP00000106750 |
| Rab3a | P63011 | 10090.ENSMUSP00000105719 |
| Rap1a | P62835 | 10090.ENSMUSP00000088174 |
| Robo2 | Q7TPD3 | 10090.ENSMUSP00000112776 |
| Ryr2 | E9Q401 | 10090.ENSMUSP00000021750 |
| Sbf1 | Q6ZPE2 | 10090.ENSMUSP00000118107 |
| Scg5 | P12961 | 10090.ENSMUSP00000024005 |
| Sh2d5 | Q8JZW5 | 10090.ENSMUSP00000101450 |
| Shank3 | Q4ACU6 | 10090.ENSMUSP00000104932 |
| Shroom2 | A2ALU4 | 10090.ENSMUSP00000098701 |
| Slc25a5 | P51881 | 10090.ENSMUSP00000016463 |
| Slc6a17 | Q8BJI1 | 10090.ENSMUSP00000029499 |
| Svop | Q8BFT9 | 10090.ENSMUSP00000050730 |
| Syt3 | O35681 | 10090.ENSMUSP00000112432 |
| Tanc2 | A2A690 | 10090.ENSMUSP00000097904 |
| Tbc1d24 | Q3UUG6 | 10090.ENSMUSP00000127005 |
| Tceb2 | P62869 | 10090.ENSMUSP00000066210 |
| Timm29 | Q8BGX2 | 10090.ENSMUSP00000058283 |
| Tmem50b | Q9D1X9 | 10090.ENSMUSP00000023686 |
| Tnks1bp1 | P58871 | 10090.ENSMUSP00000107232 |
| Tst | P52196 | 10090.ENSMUSP00000055743 |
| Uap1 | Q91YN5 | 10090.ENSMUSP00000106983 |
| Ube2v2 | Q9D2M8 | 10090.ENSMUSP00000111443 |
| Usp5 | P56399 | 10090.ENSMUSP00000041299 |
| Vdac1 | Q60932 | 10090.ENSMUSP00000099819 |
| Vps53 | Q8CCB4 | 10090.ENSMUSP00000061317 |
| Ykt6 | Q9CQW1 | 10090.ENSMUSP00000002818 |
